# MALDI imaging mass spectrometry of *N*-linked glycans on formalin-fixed paraffin-embedded murine kidney

**DOI:** 10.1007/s00216-014-8293-7

**Published:** 2014-12-02

**Authors:** Ove J. R. Gustafsson, Matthew T. Briggs, Mark R. Condina, Lyron J. Winderbaum, Matthias Pelzing, Shaun R. McColl, Arun V. Everest-Dass, Nicolle H. Packer, Peter Hoffmann

**Affiliations:** 1 Adelaide Proteomics Centre, School of Molecular and Biomedical Science, University of Adelaide, Adelaide, South Australia 5005 Australia; 2 Bruker Pty. Ltd., Preston, Victoria 3072 Australia; 3 Faculty of Science, Biomolecular Frontiers Research Centre, Macquarie University, Sydney, New South Wales 2109 Australia; 4 Chemokine Biology Laboratory, School of Molecular and Biomedical Science, University of Adelaide, Adelaide, South Australia 5005 Australia; 5 Centre for Molecular Pathology, University of Adelaide, Adelaide, South Australia 5005 Australia; 6 Institute for Photonics & Advanced Sensing (IPAS), University of Adelaide, Adelaide, South Australia 5005 Australia

**Keywords:** MALDI imaging, MALDI, Mass spectrometry, Glycans, *N*-linked

## Abstract

Recent developments in spatial proteomics have paved the way for retrospective in situ mass spectrometry (MS) analyses of formalin-fixed paraffin-embedded clinical tissue samples. This type of analysis is commonly referred to as matrix-assisted laser desorption/ionization (MALDI) imaging. Recently, formalin-fixed paraffin-embedded MALDI imaging analyses were augmented to allow in situ analyses of tissue-specific *N*-glycosylation profiles. In the present study, we outline an improved automated sample preparation method for *N*-glycan MALDI imaging, which uses in situ PNGase F-mediated release and measurement of *N*-linked glycans from sections of formalin-fixed murine kidney. The sum of the presented data indicated that *N*-glycans can be cleaved from proteins within formalin-fixed tissue and characterized using three strategies: (i) extraction and composition analysis through on-target MALDI MS and liquid chromatography coupled to electrospray ionization ion trap MS; (ii) MALDI profiling, where *N*-glycans are released and measured from large droplet arrays in situ; and (iii) MALDI imaging, which maps the tissue specificity of *N*-glycans at a higher resolution. Thus, we present a complete, straightforward method that combines MALDI imaging and characterization of tissue-specific *N*-glycans and complements existing strategies.

**Electronic supplementary material:**

The online version of this article (doi:10.1007/s00216-014-8293-7) contains supplementary material, which is available to authorized users.

**Graphical Abstract:**
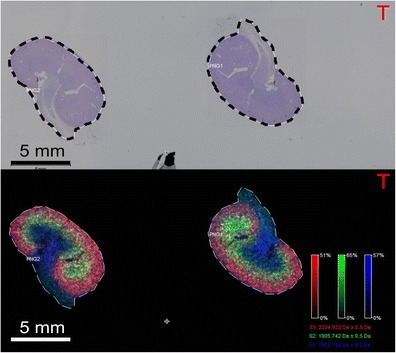
MALDI imaging MS of N-linked glycans released from formalin-fixed paraffin-embedded murine kidney sections. Ion intensity maps for (Hex)_2_(HexNAc)_3_(Deoxyhexose)_3_+(Man)_3_(GlcNAc)_2_ (*m/z* 2304.932, red), (Hex)_6_+(Man)_3_(GlcNAc)_2_ (*m/z* 1905.742, green) and (Hex)_2_(HexNAc)_2_+(Man)_3_(GlcNAc)_2_ (*m/z* 1663.756, blue)

## Introduction

The majority of mammalian secretory and membrane proteins are modified through glycosylation: the covalent linkage of polysaccharide moieties (i.e., glycans) to either serine/threonine (*O*-linked) or asparagine (*N*-linked) residues [1]. As such, glycosylation is one of the most common posttranslational modifications (PTM) [1, 2]. Global analysis of glycans and glycoproteins is currently pursued through glycomics and glycoproteomics, respectively [3]. Glycomics focuses on the isolation and characterization of glycans [3], while glycoproteomics focuses on the elucidation of information such as the identity of glycosylated proteins, glycan composition, glycosylation site, and the role of the glycoprotein in cellular function and/or dysfunction [1]. Much of the interest in the analysis of protein glycosylation stems from the observation of altered glycosylation patterns in cancer [1]. An understanding of these alterations could provide novel biomarkers of disease as well as new treatment targets for anticancer therapies [4].

There are currently a limited number of mass spectrometry (MS)-based methods available for interrogating clinical tissue samples for variations in glycosylation patterns. One reason for this has been the perception that the most common type of archival clinical sample, formalin-fixed paraffin-embedded (FFPE) tissue, was difficult to access for glycoproteomic and proteomic analyses. However, recent work has demonstrated that the inter- and intraprotein cross-links induced by formalin do not preclude the analysis of FFPE tissue proteomes by bottom-up liquid chromatography (LC)-MS/MS-based proteomics [5] and that *N*-glycosylation sites cannot only be mapped but are quantitatively conserved during the fixation process [6, 7]. The ability to analyze the glycoproteome of FFPE tissues will allow for retrospective analysis of the substantial clinical tissue archives which exist worldwide [8]. Such an approach would represent a significant step forward for glycoproteomic cancer research. Unfortunately, quantitative glycoproteomics methods are typically time-consuming, require extensive sample preparation procedures, and sacrifice spatial proteome information when the tissue is solubilized. Direct tissue analysis by MS, otherwise known as matrix-assisted laser desorption/ionization (MALDI) imaging, provides a unique solution to these issues [9]. MALDI imaging allows for the rapid collection of molecular information directly from tissue sections, without the need for homogenization, fractionation, or application of antibody-based techniques. [10, 11] Furthermore, because the MS data is spatially referenced, it can be overlaid directly with histology and tissue sections can be annotated with the spatial distribution of biological analytes [12, 13].

As mentioned above, until relatively recently, FFPE tissues were not regularly analyzed by MALDI imaging. Seminal work by Groseclose et al. in 2008 adapted the bottom-up approach for the analysis of FFPE tissues by combining antigen retrieval with spatially sensitive tryptic digestion for MALDI imaging. This approach was used to successfully classify subtypes of human lung tumor [14]. Combined with the observation that *N*-glycosylation can be analyzed in FFPE tissues, this work paved the way for glycan MALDI imaging. Powers et al. showed that *N*-glycan MALDI imaging is achievable on sections of native tissue [15]. Furthermore, Yamada et al. demonstrated access to oligosaccharides by α-amylase digestion of FFPE human intestinal tissue containing an amylopectin-rich obstruction [16]. Finally, during the preparation of this manuscript, Toghi Eshghi et al. and Powers et al. demonstrated the application of *N*-glycan MALDI imaging to FFPE murine brain tissue [17], murine kidney, gliomas, and tissue microarrays [18].

Herein, we present a complementary three-component automated sample preparation and characterization strategy developed for *N*-glycans released in an FFPE murine kidney model. The first component of this strategy was a low spatial resolution in situ MALDI profiling method for the measurement of *N*-glycans directly from specific tissue types. To confirm the detection of *N*-glycans, the second component used in-solution enzymatic release of *N*-glycans from FFPE kidney tissue followed by characterization using MALDI-time of flight (TOF)/TOF MS and LC-electrospray ionization (ESI)-IT MS/MS. The third and final components employed a higher spatial resolution MALDI imaging method to comprehensively map the tissue-specific distributions of the characterized *N*-glycans.

The presented work is complementary to existing methods and takes the next step in combining *N*-glycan MALDI imaging in kidney tissue with glycomic characterization by MS/MS. This method can potentially be used for interrogation of any FFPE-preserved tissue type to map the tissue-specific distribution of *N*-glycans.

## Material and methods

### Reagents

Glycerol-free PNGase F (P0705L, 75,000 NEB units) was purchased from New England Biolabs (Ipswich, MA, USA). 2,5-DHB matrix was purchased from Sigma-Aldrich (Steinheim, Germany) and Bruker Daltonics (Bremen, Germany). Formalin was from Sigma-Aldrich. Trifluoroacetic acid (TFA), ethanol, and NaCl were from Merck (Darmstadt, Germany). Nitrocellulose membranes (0.025 μm VSWP) for dialysis were purchased from Millipore (Cork, Ireland). Xylene was purchased from Chem-Supply (Gillman, South Australia). Indium tin oxide (ITO) slides were purchased from Bruker Daltonics, while PEN membrane slides were from MicroDissect (Herborn, Germany). GLY3 standards (see Table 1) were purchased from Prozyme (CA, USA). Unless otherwise stated, all H_2_O used was ultrapure (i.e., ≥18.2 MΩ and ≤5 ppb TOC).

**Table 1:** *N*-glycan standard mixture (GLY3) used for calibration of MALDI-TOF/TOF MS instrument

*N*-glycan composition	[M]	[M+Na]^+^	Concentration	Company
Man_5_GlcNAc_2_	1234.4333	1257.4225	1 pmol/μL	Prozyme, CA, USA
Man_3_GlcNAc_5_	1519.5659	1542.5551		
Man_3_Gal_4_GlcNAc_6_	2370.8565	2393.8457		

### Tissue sectioning

Murine kidney tissue was scavenged from mice sacrificed as part of experiments with full ethics approval and in accordance with the policies of the University of Adelaide Ethics Committee. Formalin was diluted 1:9.25 in 1× PBS, and tissues were immersed in this solution overnight at 4 °C. The tissue was then removed from the formalin, rinsed with deionized H_2_O, and stored in 70 % ethanol (EtOH) before processing with a Leica TP 1020 processor (Leica Biosystems, North Ryde, Australia). The protocol was as follows: 70 % EtOH for 5 min, 80 % EtOH for 2 h, 95 % EtOH for 2 h, 3× 100 % EtOH for 2 h each, 2× xylene for 2 h each, and 2× paraffin for 2 h each. A Leica EG 114OH embedder (Leica Biosystems) was used to create paraffin-embedded tissue blocks. FFPE blocks were sectioned (6 μm thick) on a Microm HM 325 microtome (Zeiss, Göttingen, Germany) and water bath mounted (39 °C) onto ITO slides (Bruker Daltonics) or PEN membrane slides (MicroDissect). Slides were left to dry at 37 °C for 1 h prior to storage at 4 °C.

### In-solution PNGase F digestion and matrix preparation and MALDI-TOF/TOF MS

Tissue on PEN membrane slides (*n* = 3) was used for manual microdissection and processing as described previously [19, 20], with modifications. Briefly, following a 5 min 60 °C heating block incubation, 90 s xylene wash, and 60 s EtOH wash, the kidney tissue sections were isolated by manual microdissection and deposited into separate microvials. The tissue was incubated (2 × 5 min) in 10 mM NH_4_HCO_3_, and the solution was removed and replaced with 10 mM citric acid (pH 6) and heated at 98 °C for 45 min. The tissue was washed twice with 10 mM NH_4_HCO_3_. Glycerol-free PNGase F (2 μL, nondialyzed) was added to the FFPE tissue in a 40 μL total reaction volume (with 25 mM NH_4_HCO_3_). The tissue digest was left overnight at 37 °C. The reaction volume was then spotted (1 μL) onto an MTP-384 800 μm AnchorChip target (Bruker Daltonics) and allowed to dry at room temperature. A custom *N*-glycan standard (GLY3 standard, see Table 1) was spotted (0.5 μL) onto calibrant AnchorChip spots.

The sample was overlaid using a modified EZYprep deposition method [21]. Briefly, 10 mg/mL 2,5-DHB in 0.1 % TFA and 1 mM NaCl was deposited in multiple layers using custom settings [0.075 mL/min flow rate, 4 psi N_2_ pressure, 85 °C capillary temperature, 8 passes, 800 mm/min] for a TM sprayer instrument (HTX Technologies, Carrboro, NC, USA). MS data was acquired on an ultrafleXtreme MALDI-TOF/TOF instrument operated through an autoXecute method in flexControl (v3.4, Bruker Daltonics). External calibration was performed using the GLY3 standard (Table 1). MS spectra were processed in flexAnalysis. Peaks were picked using the SNAP algorithm with an averagine approximation and signal to noise (*S*/*N*) >3.

### *N*-glycan purification and LC-ESI-ion trap MS and MS/MS

The released *N-*linked glycans were reduced and desalted according to previously described methods [22]. After acidification with 100 mM NH_4_COOH pH 5 (10 μL) for 60 min at room temperature, the samples were dried in a vacuum centrifuge and were reduced with 20 μL of 1 M NaBH_4_ in 50 mM KOH at 50 °C for 3 h. The reduction was quenched with 1 μL glacial acetic acid, and the *N*-linked glycan alditols were desalted as described below. The released glycans were desalted using cation-exchange columns comprising 30 μL AG50W-X8 cation-exchange resin (Bio-Rad, Hercules) packed on top of a C18 StageTip Frit. Residual borate was removed by the addition of methanol (3×) and dried under vacuum. The glycans were further purified by tips using porous graphitized carbon slurry manually packed onto C18 StageTip Frits as previously described [22]. The glycans were resuspended in 10 μL of Milli-Q water and subjected to porous graphitized carbon (PGC)-LC-ESI MS/MS separation and analysis. *N*-linked glycan alditols were separated using an Agilent 1100 capillary LC (Agilent Technologies, Santa Clara, CA) and analyzed using an Agilent MSD, three-dimensional ion trap XCT mass spectrometer coupled to the LC. Separation was performed on a Hypercarb PGC column (3 μm particle size, 100 × 0.18 mm, Hypercarb, Thermo Scientific) across an 85 min gradient with a constant flow rate of 2 μL/min using a linear gradient up to 45 % (*v*/*v*) CH_3_CN in 10 mM NH_4_HCO_3_. ESI MS was performed in negative ion mode with two scan events: MS full scan with mass range 100–2000 *m*/*z* and data-dependent MS/MS scan after collision-induced dissociation (CID) of the top two most intense precursor ions.

### PNGase F dialysis and tissue section antigen retrieval

Neat PNGase F enzyme (20 μL for profiling, 40 μL for MALDI imaging) was dialyzed on a nitrocellulose membrane against water for 2 h. Tissue sections were rehydrated using a standard procedure for citric acid antigen retrieval (CAAR) [20]. Briefly, the tissue sections were heated at 60 °C for 1 h and washed in 100 % xylene (2 × 5 min) and 100 % ethanol (2 × 2 min). Sections were washed in 10 mM NH_4_HCO_3_ (2 × 5 min) followed by a 10 min pulse boil in 10 mM citric acid (pH 6.0) followed by heating at 98 °C for 30 min on a heating block. Finally, the tissue sections were immersed twice in 10 mM NH_4_HCO_3_ (1 min) and dried at room temperature in a humid chamber.

### In situ PNGase F digestion and matrix deposition

NH_4_HCO_3_ (25 mM, pH ∼8.2) was added to the dialyzed PNGase F to a total volume of 100 μL for profiling or 200 μL for MALDI imaging. PNGase F was printed onto retrieved tissue sections—750 nL at 1300 μm center to center spacing for profiling or 30 nL at 250 μm spacing for MALDI imaging—using a ChIP-1000 (Shimadzu, Japan). Buffer control arrays (25 mM NH_4_HCO_3_) were printed using the same conditions on adjacent sections. Tissue sections were incubated overnight at 37 °C in a humid chamber, and GLY3 standard was manually spotted (0.5 μL) on an adjacent section. 2,5-DHB (10 mg/mL for profiling or 20 mg/mL for MALDI imaging) in 0.1 % TFA and 1 mM NaCl was sprayed onto prepared tissues using a TM sprayer. Instrument-specific settings are as follows: 16 passes, 0.05 mL/min flow rate, 4 psi N_2_ pressure, 65 °C capillary temperature, and 800 mm/min.

### MALDI-TOF/TOF mass spectrometry and histological staining

MS data was acquired using an ultrafleXtreme MALDI-TOF/TOF mass spectrometer (Bruker Daltonics) controlled by flexControl (v3.4, Bruker Daltonics) and flexImaging (v4.0, Bruker Daltonics). Instrument-specific settings were as follows: 800–4500 *m*/*z* range, 700 Da matrix suppression, 2 kHz laser repetition rate, and 2.5 GS/s.

(i) *Profiling:* Method-specific settings were as follows: 70 % laser power and 2694 V detector gain. A total of 10,000 shots (random walk) were acquired at each treatment position and the calibration spots for each of the four profiling replicates.
(ii) *Imaging:* Method-specific settings were as follows: 75 % laser power and 2698 V detector gain. A total of 1000 shots were acquired at each position with no walk. Data was acquired by oversampling from an arbitrary array with center to center acquisition distance of 100 μm overlaid onto the 250 μm PNGase F array. The MALDI-TOF/TOF instrument was externally calibrated using the included GLY3 standard spots prior to MALDI imaging.

Following analysis, the matrix was eluted using 70 % EtOH and tissue sections were hematoxylin and eosin (H & E)-stained using a standard protocol.

### Data analysis—MALDI profiling and MALDI imaging

(i) *Profiling:* Baseline-subtracted MS spectra were visualized in flexImaging (Bruker Daltonics) using data reduction to 20,000 points and root mean square (RMS) normalization. External calibrant spectra were internally recalibrated using a quadratic fit to the GLY3 mass control list in flexAnalysis (v3.4, Bruker Daltonics). Of two calibrant spots, the best calibration was copied to all MS spots (buffer control and PNGase F) for that respective tissue section. Calibration was repeated for all sections (*N* = 4). All peak lists were exported using flexAnalysis into ASCII text files. A custom R [23] script was written to further process these peak lists. Text files were combined and peaks were grouped using a linkage distance of 0.1 *m*/*z*. Peak groups occurring in ≥2 counts in the PNGase F spectra and ≤1 count in the buffer control spectra were considered. Abundance weighted mean (AWM, weighted by *S*/*N*) *m*/*z* was calculated for each of these peak groups.
(ii) *MALDI imaging:* Data was analyzed by SCiLS Lab (version 2014a, SCiLS GmbH, Bruker Daltonics) or by exporting peak lists from flexAnalysis (TopHat baseline subtraction and SN >3) and in-house R [23] processing for difference in proportions statistic (DIPPS) analysis. For the SCiLS analysis, raw data was loaded and reduced to 50,000 points per spectrum. Data was preprocessed by baseline subtraction and normalization to total ion current (TIC). Peaks were picked and aligned, and the preprocessed data was used for spatial segmentation (bisecting *k*-means with correlation distance) as well as a random initialization probabilistic latent semantic analysis (pLSA) with 10 components. Minimal interval width was set to 0.5 Da for both analyses. For DIPPS analysis in R, peaks were grouped using a linkage distance of 0.1 Da, and weighted mean *m*/*z* values were calculated for each peak group using *S*/*N* ratios for weights. Only peak groups (i.e., analytes) occurring in at least 100 spectra were considered. The difference in proportions of occurrence (between PNGase F-treated and control regions), known as DIPPS statistics, was calculated. A high DIPPS value (close to 1) for a given peak group indicates that peaks in that peak group occur in a high proportion of spectra from PNGase F-treated regions and a low proportion of spectra from control regions. DIPPS maps were created which show the spatial distribution of a selected number of peak groups (with high DIPPS) by counting the number of the selected peak groups that exhibit peaks in each spectrum and plotting those counts spatially. The number of peak groups selected for inclusion can be data independent (i.e., user selected) or data dependent (i.e., based upon a heuristic cut-off). Log ion intensity maps were also created for peak groups with high DIPPS. The development and use of the DIPPS method are further described in an unpublished work [24].

## Results and discussion

Formalin-fixed tissue sections are unique in that the proteome is cross-linked extensively and is immobilized as a result. This situation is advantageous for glycomic MALDI imaging because glycosidases can be applied directly to the tissue to release *N*-glycans. These *N*-glycans can then be analyzed as a relatively pure population without the presence of contaminating endogenous analytes such as lipids and peptides, which would be present in native tissue sections. In order to leverage this advantage and to address both of the typical in situ MS measurement modes, the work presented in this manuscript developed both large droplet profiling and spatially resolved MALDI imaging approaches for *N*-glycan analysis.

**Fig. 1:**
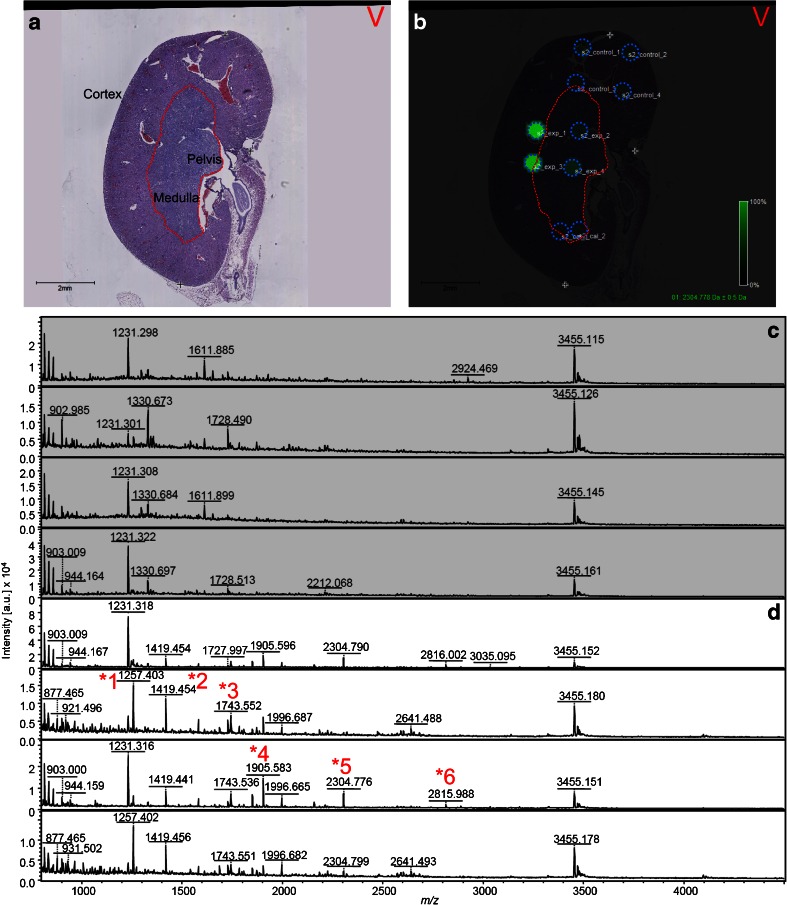
In situ MS profiling of *N*-linked glycans released from formalin-fixed murine kidney sections using dialyzed PNGase F. Four replicate formalin-fixed murine kidney tissue sections were treated with antigen retrieval followed by ChIP-1000 printing of PNGase F or buffer control. An *N*-glycan calibrant mixture was spotted onto a separate part of the section. Tissue was overlaid with 2,5-DHB in 0.1 % trifluoroacetic acid and 1 mM NaCl using a TM sprayer instrument and MS spectra were acquired using a MALDI-TOF/TOF. **a** Hematoxylin and eosin (H & E)-stained murine kidney section annotated with the cortex, medulla, and pelvic regions. Cortex-medulla interface marked by a *red dotted line* (see Oetjen et al. [25]). **b** Overlay of H & E and profiling spots (*blue dotted outline*) treated with PNGase F (*middle*), buffer control (*top*), and calibrant spots (*bottom*). Ion intensity map in **b** is for mass filter 2304.778 (raw data loaded, baseline subtracted, data reduced to 20,000 points per spectrum, RMS normalization). The intensity map shows definitive signal in PNGase F-treated spots, with tissue-specific differences clear between spots localized to the cortex and those in the medulla. **c**, **d** The 4× control and 4× PNGase F MS spectra, respectively, for this section. Spectra were exported (flexAnalysis) and formatted in MS PowerPoint and InkScape (www.inkscape.org). MS spectra show clear differences in peak number, with the PNGase F spots containing multiple unique masses. *Numbers with asterisks* indicate *N*-glycan candidates identified by LC-MS/MS—these are also annotated in Table 2

To achieve profiling and demonstrate coarse tissue-specific MS measurement of the observable *N*-glycan population in FFPE tissue, four replicate FFPE murine kidney tissue sections were treated with antigen retrieval, and large droplets of glycerol-free PNGase F were printed onto the sections and incubated overnight at 37 °C. To minimize the amount of salt deposited in situ and maximize signal quality, glycerol-free PNGase F was dialyzed against ultrapure water prior to resuspension in NH_4_HCO_3_. Following incubation, the droplet arrays were prepared with 2,5-DHB. Unfortunately, typical dried droplet preparations with 2,5-DHB cause problems for both manual and automated MS acquisitions: this is predominantly due to the size and heterogeneity of the crystals (see examples of crystal shape from previous experiments in the Electronic Supplementary Material (ESM) Fig. S1a), which leads to mass errors, “sweet spot” formation, and a concomitant decrease in signal reproducibility [21]. As a result, we opted to avoid printing matrix onto the profiling spots and instead adapted our previously published EZYprep method for use on a TM sprayer instrument (HTX) [21]. EZYprep depositions involve nebulization of matrix onto pre-prepared sample, a process that produces finer 2,5-DHB crystals (see ESM Fig. S1b), which in turn reduces sweet spot formation and improves mass accuracy, reproducibility, and signal intensity. As such, a TM sprayer was used to deposit multiple 2,5-DHB layers, followed by MALDI-TOF/TOF MS. Figure 1a shows a H & E stain for one of the four MALDI profiling sections, with the major histological regions, cortex, medulla, and pelvis annotated, and the border between the cortex and medulla indicated with a red dotted line [25]. Figure 1b highlights the molecular profiling locations overlaid with the H & E stain. These locations included four NH_4_HCO_3_ controls (Fig. 1b, top), four PNGase F spots (Fig. 1b, middle), and two *N*-glycan calibrant spots (see Table 1) (Fig. 1b, bottom). The RMS normalized intensity for *m*/*z* 2304.85 (AWM *m*/*z*) is also presented in Fig. 1b. For this example *m*/*z* and multiple others, the buffer control spots showed virtually no normalized signal intensity. This was in contrast to the PNGase F profiling spots which showed definitive signal and were able to discern the first tissue-specific differences between the cortex and medulla of the kidney section (see green ion intensity in Fig. 1b).

Figure 1b clearly shows that the intensity differences across the four PNGase F spots correspond to the internal structure of the kidney. For example, *m*/*z* 2304.85 could be found in the cortex but not in the medulla (green ion intensity signal in Fig. 1b). The MS spectra for the tissue in Fig. 1a, b are provided in Fig. 1c, d for the buffer control and PNGase F profiling spots, respectively. MS spectra for the remaining three replicate tissue sections are provided as ESM Fig. S2.

To isolate those peaks that occur predominantly in the PNGase F profiling spots and, therefore, identify all the detectable *N*-glycan candidates, externally calibrated peak lists for all 32 spots (control and PNGase F) were grouped, and the occurrence of all peak groups (i.e., analytes) in both control and PNGase F spots was counted. In total, 79 peaks existed in ≥2 of the PNGase F spots and ≤1 of the buffer control spots and were larger than *m*/*z* 1000. Making this threshold for *N*-glycan candidate status more conservative (by requiring ≥4 counts in PNGase F spots) decreased the total to 43 *N*-glycan candidates. As an example, *m*/*z* 2304.85 could be detected in 15/16 of the PNGase F-treated profiling locations and at a low *S*/*N* in 1/16 buffer control locations. The few instances of overlap in this manner were attributed to the possible delocalization of PNGase F from an adjacent treated spot.

**Fig. 2:**
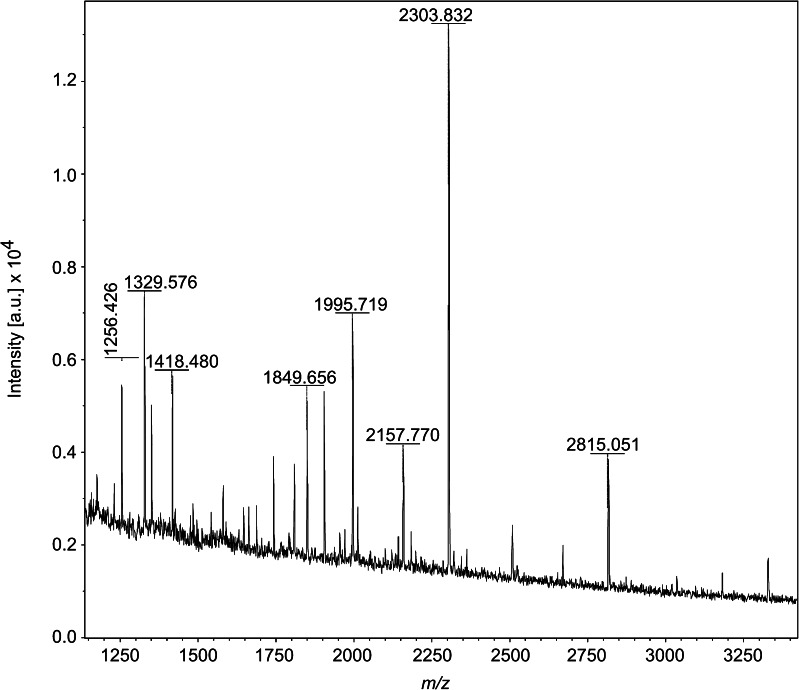
MALDI-TOF/TOF MS and MS/MS of *N*-glycans released in-solution from formalin-fixed murine kidney sections. MS profile obtained from *N*-glycans enzymatically released (PNGase F) in-solution from FFPE kidney tissue treated with antigen retrieval. Spectrum was acquired on a MALDI-TOF/TOF MS instrument in reflectron positive ion mode and peaks were picked (no further processing). Formatting in MS PowerPoint and InkScape (www.inkscape.org). Multiple unique masses can be visualized in the PNGase F-treated tissue supernatant. (see corresponding *m/z* of potential *N*-linked glycans in Table 2)

To confirm the identity of these candidates, *N*-glycans were extracted directly from tissue sections for characterization. This type of process is straightforward and allows the characterization of *N*-glycans from relatively small amounts of tissue sample [26]. Sections of FFPE murine kidney tissue (*N* = 3) were manually microdissected, treated with antigen retrieval, and incubated overnight with PNGase F (two of three sections). The experimental reaction supernatants, in addition to the tissue only (one of three sections), PNGase F only, and blank controls, were analyzed on an ultrafleXtreme MALDI-TOF/TOF. The MS profiles for tissue only, PNGase F only, and blank controls contained only a few peaks (see ESM Fig. S3). In stark contrast, the MS spectra for both PNGase F-tissue reaction supernatants contained multiple high *S*/*N* peaks (see Fig. 2). Interestingly, when comparing the MS profiles for the in situ MALDI profiling and on-target MALDI-TOF/TOF, an approximate 1 Da mass discrepancy was noted for multiple *N*-glycans. Additionally, the isotopic profiles of these *N*-glycans changed (see ESM Fig. S4). For example, the isotopic profile of [M+Na]^+^ on-target indicated the overlap of two species at roughly 2303.8 and 2304.8. Two possible explanations exist for this observation. Either there are two different *N*-glycan species with a difference in mass of roughly 1 Da, or there are two forms of the same *N*-glycan. In the latter case, the same *N*-glycan would exist with a glycosylamine (e.g., 2303.8) or free reducing end (e.g., 2304.8) at the HexNAc C1 position.

**Fig. 3:**
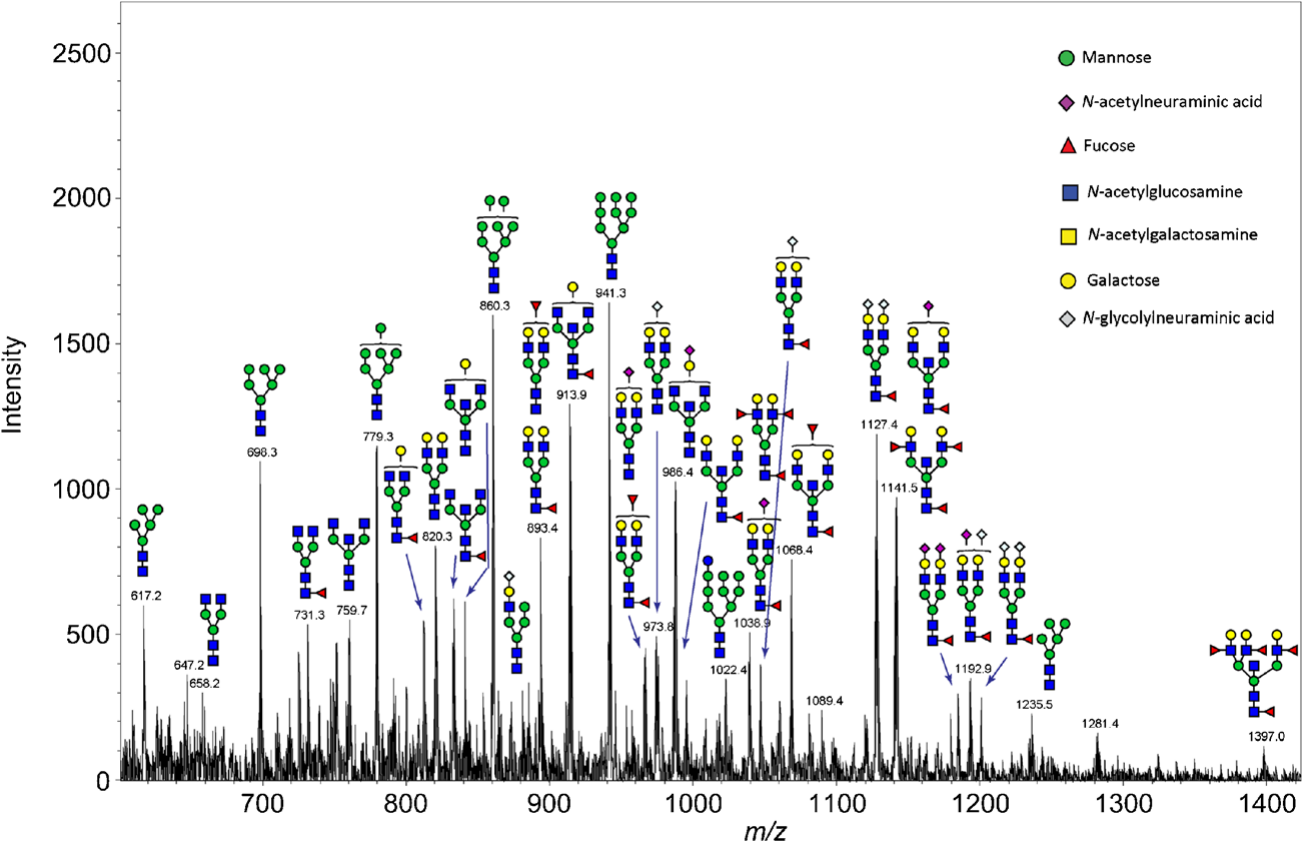
Ion chromatogram annotated with structure proposals for *N*-glycans identified by ESI-ion trap MS/MS. *N*-glycans released in-solution from formalin-fixed murine kidney tissue using PNGase F were reduced, desalted, and purified on a carbon column prior to porous graphitic column chromatography coupled online to an ESI-ion trap (IT) system. MS scan above is annotated with the structure proposals that resulted from MS/MS structure profiling of the eluate

To further characterize the *N*-glycan mixture, the reaction supernatant was reduced, desalted, and purified on in-house packed carbon columns prior to graphitic column chromatography coupled online to an ESI-ion trap. MS/MS resulted in the identification of 34 *N*-glycan peaks. Figure 3 shows an ion chromatogram annotated with the structure proposals for *N*-glycans identified by MS/MS. As can be seen, the structure proposals include a mixture of oligo-mannose, complex and hybrid *N*-glycans (example MS/MS spectra available as ESM Fig. S5). All of the measured ions were doubly negatively charged, with the exception of [M−H]^−^: 1235.5. Several acidic *N*-glycans with terminal *N*-glycolylneuraminic acid and *N*-acetylneuraminic acid were also identified during this analysis. The heat-induced antigen retrieval was used to ensure that the sample preparation conditions for the extracted *N*-glycans matched the in situ MALDI profiling. However, the heating step was also expected to reduce the likelihood of observing sialylated *N*-glycans. As such, it cannot be ruled out that milder rehydration conditions could allow the detection of more sialylated *N*-glycan species by LC-MS/MS, and method modifications to achieve this warrant further investigation.

Table 2 provides a combined list of the *N*-glycan candidates which were found by MALDI profiling and further identified and characterized by LC-MS/MS (Fig. 3). Included in the table are the buffer control and PNGase F counts, the AWM [M+Na]^+^ for each *N*-glycan, its calculated [M+Na]^+^, the associated error as well as the calculated and measured [M−2H]^2−^ for the LC-MS/MS analysis. The proposed compositions and structures are also provided as well as the calculated [M+Na]^+^ from a previous study, if available [27].

**Table 2:**
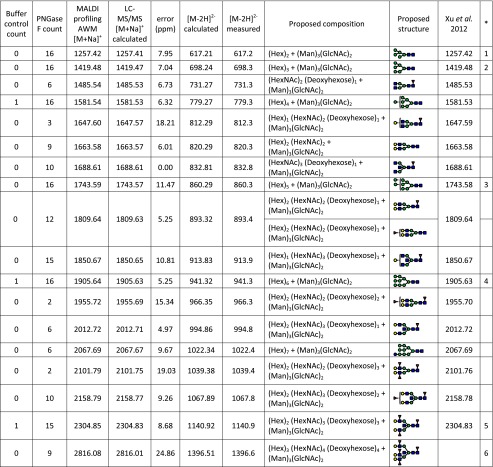
*N*-glycan candidates detected by large droplet profiling and characterized by LC-ESI-ion trap MS/MS. Provided in this table are the occurrence counts for *N*-glycan candidates found in formalin-fixed murine kidney by in situ profiling (buffer control + PNGase F spots), their sodiated abundance weighted mean mass (profiling AWM [M+Na]^+^), the calculated sodiated mass ([M+Na^+^]^+^ calculated), error (ppm) between the profiling and calculated sodiated masses, the calculated doubly negatively charged mass ([M−2H]^2−^ calculated), the measured doubly negatively charged mass ([M−2H]^2−^ measured), proposed composition, the proposed structure, and the calculated sodiated mass from a previous publication [27]

The column with the heading “*” indicates those *N*-glycans annotated in Fig. 1 MS spectra

Mass correlations to the *N*-glycans in [27] are provided in ESM Table S1. The full list of *N*-glycan candidates as well as the *N*-glycans identified by LC-MS/MS is provided in ESM Table S2. In total, 23 [M+Na]^+^ values from the profiling analysis could be correlated to the calculated [M+Na]^+^ values reported in [27]: this amounts to 67 % of the previously reported glycan species. Identity could be assigned to 18 *N*-glycan candidates in this study (Table 2). It should be noted that several of the *N*-glycans not detectable during MALDI profiling were acidic or high-mannose type (see Fig. 3). As such, additional method developments to allow the detection of these *N*-glycans by MALDI profiling would be beneficial (e.g., derivatization), particularly for application to tumor tissues where acidic *N*-glycans are known to be significantly overrepresented.

To increase the spatial resolution of analysis, the profiling method was miniaturized to allow for MALDI imaging on the FFPE kidney tissue. Printing of PNGase F onto antigen-retrieved tissue followed by overnight incubation at 37 °C and spray deposition of 2,5-DHB was found to provide consistent *N*-glycan signal across entire tissue sections. This method was similar to that previously published by Toghi Eshghi et al. [17], with the exception that the current method is completely automated, uses contactless printing (i.e., does not touch the tissue surface) of reagents, and employs substantially reduced buffer salt concentration (25 mM versus 1 M) for PNGase F digestion. In general, the most important sample preparation requirements seemed to be the PNGase F buffer pH and the homogeneity of the final deposited matrix layer, which is ultimately affected by reproducibility of the sample preparation method and the presence of contaminants such as salts and polymers.

**Fig. 4:**
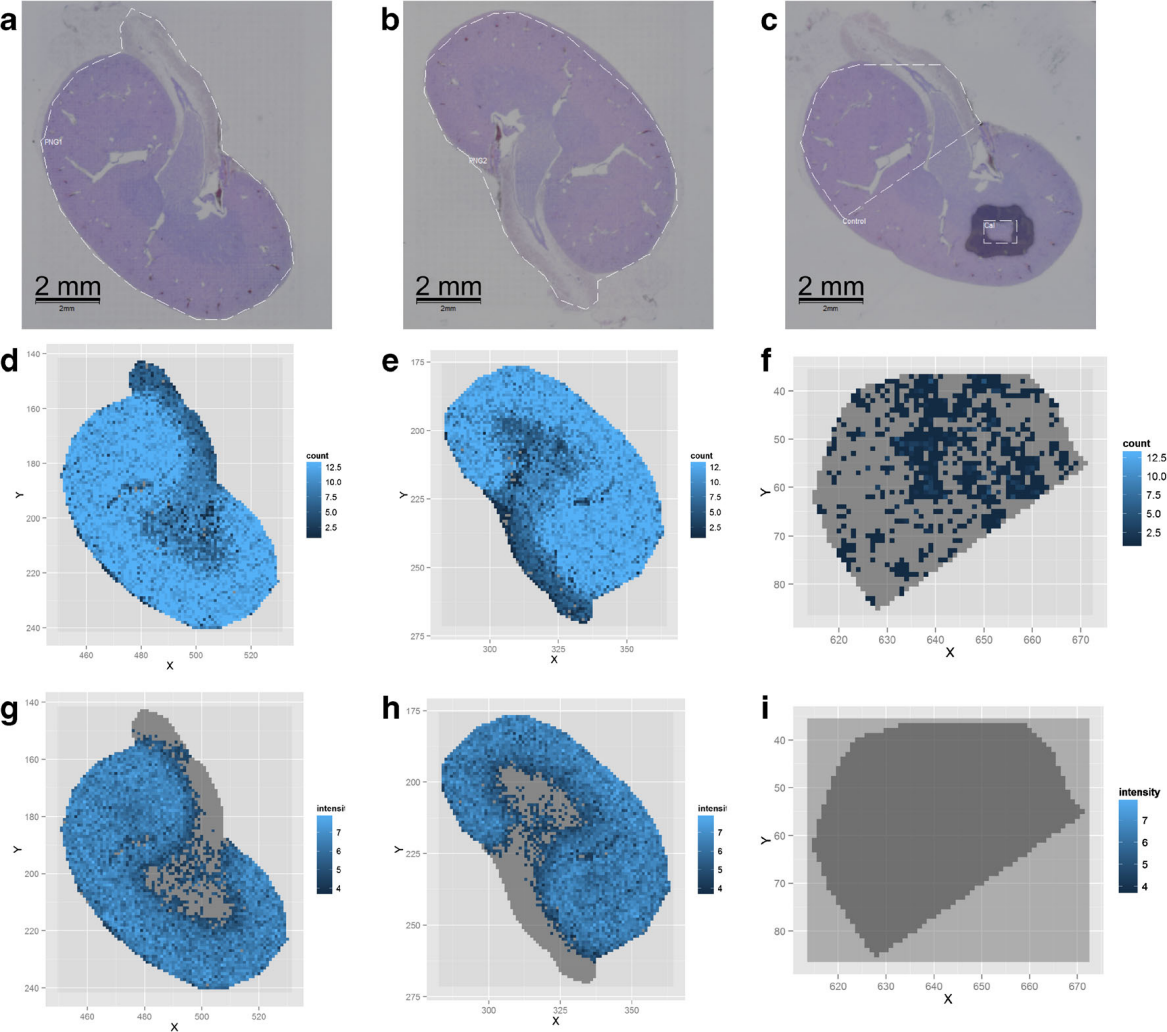
MALDI imaging MS of *N*-linked glycans released from formalin-fixed murine kidney sections. Formalin-fixed murine kidney sections were treated with citric acid antigen retrieval prior to printing of 30 nL/spot dialyzed PNGase F or buffer control arrays with 250 μm spacing onto the tissue. 2,5-DHB (20 mg/mL) was sprayed onto the tissue sections, and MS spectra were acquired by oversampling at 100 μm intervals using a MALDI-TOF/TOF MS instrument. Hematoxylin and eosin stained tissue sections are shown for PNGase F-treated (**a**, **b**) and buffer control-treated (**c**) regions, respectively. **d**–**f** DIPPS maps of the 13 *m*/*z* values with the highest DIPPS score (i.e., highest proportion in PNGase F-treated regions as compared to control) for the PNGase F (**d**, **e**) and buffer control (**f**) regions, respectively. **g**–**i** The log ion intensity maps for one of these 13 *m*/*z* values, in this case 2304.909, for the PNGase F (**g**, **h**) and buffer control (**i**) regions. Both DIPPS maps and log ion intensity maps show increased counts/intensity in the cortex of the kidney

Duplicate FFPE kidney sections were prepared for *N*-glycan MALDI imaging. MS data was acquired by oversampling printed PNGase F arrays. Figure 4a–c shows the H & E stains for the duplicate PNGase F-treated sections and the buffer control-treated section, respectively (measurement regions indicated by a dashed white outline). A twofold approach was taken to evaluate the acquired data in terms of (1) finding masses unique to PNGase F-treated regions (as compared to buffer control) and (2) the ability of the MALDI imaging data to map histological regions of the kidney.

To address the unique masses in PNGase F-treated regions, DIPPS were calculated. High values of DIPPS highlight masses which show a high proportion of occurrence in the PNGase F-treated region and a lower proportion of occurrence in control, which would be expected to be *N*-glycans. Using a data-dependent heuristic cut-off for the DIPPS value, 13 *m*/*z* values were identified as optimal for characterizing the PNGase F-treated tissues. Figure 4d–f contains the DIPPS map representation of these 13 *m*/*z* values for the two PNGase F-treated tissue sections and buffer control section, respectively. These DIPPS maps count the occurrence of the 13 selected peak groups at each spatial location in the MALDI imaging data set. The DIPPS maps for the duplicate sections indicated that the majority of masses seem to be cortex specific, as this region represents the majority of the tissue. The DIPPS map for the buffer control-treated tissue (Fig. 4f) shows almost no occurrence of these peak groups. Of the 13 values isolated by DIPPS, nine values matched to those identified by LC-MS/MS in Table 2. The AWM *m*/*z* values for these nine *N*-glycans were 1257.473, 1905.697, 1743.643, 1581.587, 1850.730, 2304.909, 2158.844, 1419.532, and 1809.69. If, rather than considering the top 13, we consider the top 26 peak groups as ranked by DIPPS, the number of matches to LC-MS/MS identified *N*-glycans increases to 16 (additional *m*/*z* values—1663.632, 2816.115, 1485.591, 1688.663, 2012.773, 1647.635, and 2067.747—see Table 2).

Comparing the initial nine *m*/*z* values to their calculated values in Table 2, the average mass error for these *N*-glycans was 38.7 ppm (from calculated sodiated masses). The proximity of the AWM [M+Na]^+^ values to their calculated values indicated that the automated sample preparation was sufficiently homogeneous across multiple analysis regions so as to minimize mass deviations caused by matrix heterogeneity. It should be noted that external calibration on an adjacent tissue is not ideal for precious tissues. As such, internal calibrants or a higher mass accuracy MS instrument would constitute important additions to any future *N*-glycan MALDI imaging workflow [18, 19].

Figure 4g–i provides the log ion intensity maps of AWM [M+Na]^+^: 2304.909 for the two PNGase F-treated sections and buffer control section, respectively. As this example ion intensity map shows, the spatial distribution of *N*-glycans, in this case to the cortex, can be clearly visualized using this MALDI imaging method. Figure S6 in the ESM presents the remaining ion intensity maps which were above the DIPPS cut-off threshold for one of the PNGase F-treated replicate kidney sections.

**Fig. 5:**
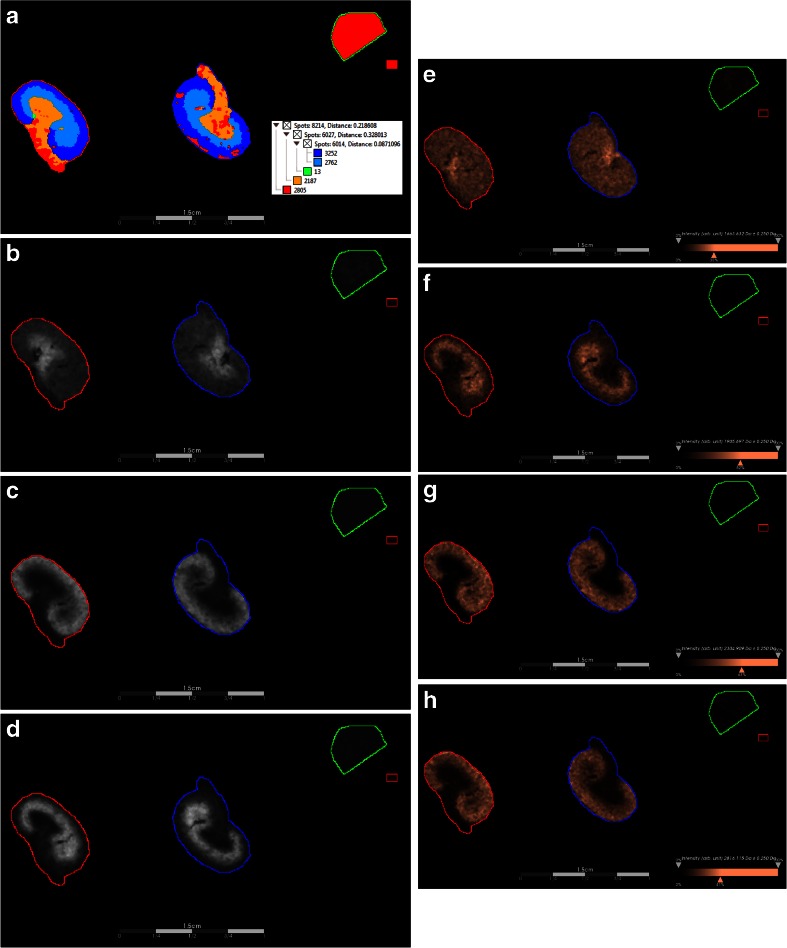
MALDI imaging MS of *N*-linked glycans released from formalin-fixed murine kidney sections. Formalin-fixed murine kidney sections were treated with antigen retrieval prior to printing of 30 nL/spot dialyzed PNGase F or buffer control arrays with 250 μm spacing. 2,5-DHB (20 mg/mL) was sprayed onto the sections and MS spectra were acquired by oversampling at 100 μm intervals using a MALDI-TOF/TOF MS instrument. Data was loaded raw into SCiLS, preprocessed for baseline subtraction and normalization to total ion current (TIC) prior to segmentation analysis (maximum processing mode, interval width of 0.5 Da, strong smoothing) and pLSA (10 component, interval width of 0.5 Da, random initiation). Regions are outlined for PNGase F regions 1 (*red*) and 2 (*blue*), as well as control (*green*) and calibrant (*small red box*) regions. **a** The segmentation map for the entire data set, which discriminates between the cortex and medulla/pelvis of the kidney at the segmentation levels selected (see *inset*). **b**–**d** Components 1, 2, and 8 from the pLSA analysis, which also discriminate the same regions of the tissue. **e**–**h** Ion intensity maps for (Hex)_2_ (HexNAc)_2_ + (Man)_3_(GlcNAc)_2_ (*m*/*z* 1663.632), (Hex)_6_ + (Man)_3_(GlcNAc)_2_ (*m*/*z* 1905.697), (Hex)2(HexNAc)_3_(Deoxyhexose)_3_ + (Man)_3_(GlcNAc)_2_ (*m*/*z* 2304.909), and (Hex)_3_(HexNAc)_4_ (Deoxyhexose)_4_ + (Man)_3_(GlcNAc)_2_ (*m*/*z* 2816.115). Intensity scales are autocorrected for these intensity maps (with weak denoising) and *scale bars* are included

In order to demonstrate data-dependent visualization of histological regions within the kidney tissue, the full data set was loaded into the SCiLS software platform and processed for both spatial segmentation analysis [28] and pLSA [29]. First, the MALDI imaging data was preprocessed and segmented. Figure 5a shows the result of this spatial segmentation. The levels selected in the segmentation dendogram allowed delineation of the cortex and medulla/pelvic regions of the kidney while also separating the PNGase F-treated and buffer control regions. To address the same question in a different manner, random initialization pLSA with 10 components was applied to the same data set. Figure 5b–d presents three histologically relevant components from this analysis, which overlay with the medulla/pelvis (Fig. 5b) as well as the cortex (Fig. 5c, d) of the kidney sections analyzed. Figure 5e–h shows the ion intensity maps for four individual [M+Na]^+^ values which correspond to (Hex)_2_ (HexNAc)_2_ + (Man)_3_(GlcNAc)_2_ (*m*/*z* 1663.632, Fig. 5e), (Hex)_6_ + (Man)_3_(GlcNAc)_2_ (*m*/*z* 1905.697, Fig. 5f), (Hex)_2_(HexNAc)_3_(Deoxyhexose)_3_ + (Man)_3_(GlcNAc)_2_ (*m*/*z* 2304.909, Fig. 5g), and (Hex)_3_(HexNAc)_4_ (Deoxyhexose)_4_ + (Man)_3_(GlcNAc)_2_ (*m*/*z* 2816.115, Fig. 5h). A consistent, spatially distributed signal was present for all four of these *N*-glycans across the kidney tissue.

With respect to tissue specificity, the four *N*-glycans listed above localized to the medulla/pelvis (Fig. 5e) and cortex (Fig. 5f–h). As expected, no normalized intensity was visible in the buffer control region. Importantly, the observation of duplicate reproducibility and the ability to clearly annotate histologically relevant spatial distributions for *N*-glycans demonstrate the utility of the presented method for molecular imaging.

To further improve the results presented here and detect more of the LC-MS/MS-identified *N*-glycans by MALDI imaging, several strategies could augment the presented method. Firstly, additional steps which promote protein denaturation (e.g., reduction/alkylation) could be employed to improve PNGase F enzyme efficiency [17]. Predigestion derivatization steps could also be employed to allow routine detection of sialylated *N*-glycans in MALDI imaging [30]. These kinds of predigestion treatments are very suitable for FFPE tissues, as the proteome is immobilized by cross-links, and as such, tissue sections can be completely immersed in reagent solutions without risking analyte delocalization and loss of spatial information. In addition to signal improvement, the glycan standard created for this study could be implemented as an internal calibrant to improve mass accuracy even further and improve confidence in correlations between MALDI imaging and LC-MS/MS. [19] Finally, *N*-glycan analysis in MALDI imaging could also be attempted in the negative mode. Although the ionization intensity might be lower than positive mode, valuable MS/MS fragmentation data could be acquired similar to that observed in the LS-ESI-MS/MS analysis. Additionally, negative mode analysis of native *N*-glycan anion adducts [31–33] can be well characterized by MALDI. However, work from the same author shows that derivatization of sialic acids can also be used to detect and to distinguish between α 2-3 and α 2-6 linkages in MALDI [30].

The sum of the presented data indicates that the methods developed in this study allow for spatial annotation of *N*-glycans on FFPE tissue sections as well as straightforward in-solution *N*-glycan release for downstream characterization by LC-MS/MS. The use of automated sample preparation instruments for PNGase F printing and matrix spraying presents a clear advantage over previous methods [17].

In the context of the work published during the preparation of the current manuscript, Powers et al. also presented *N*-glycan MALDI imaging data for murine kidney [18]. This work matched 28 *N*-glycan values from murine kidney to those available from the Consortium for Functional Glycomics (CFG) [18]. The method presented here is advantageous as it implements commercially available PNGase F rather than expressed and purified enzyme used previously [18]. This makes the method more accessible to MALDI imaging groups. Furthermore, 14 of the previously detected *N*-glycan masses were characterized by LC-MS/MS and matching candidate masses were detected for 23 of the 28 *N*-glycans [18]. The work presented here therefore presents a comprehensive MALDI imaging and glycomic characterization of *N*-glycans which complements a previous work on kidney tissue.

As mentioned above, further method development will aim to improve the sensitivity and mass accuracy for *N*-glycan MALDI imaging. Ultimately, the methods presented will be applied to *N*-glycan profiling in human cancer tissues [18]. Ovarian cancer would benefit from the application of the methods developed here for two reasons: firstly, the inadequate specificity of the glycoprotein CA125 as a biomarker for early stage diagnosis [34] and, secondly, extensive reporting of altered glycan patterns for this disease. For example, glycan alterations have been detected in the serum of patients with advanced ovarian cancer, including increased levels of sialyl Lewis x (sLeX) and core-fucosylation of agalactosylated biantennary glycans (FA2) [35].

Future analyses employing *N*-glycan MALDI imaging will initially focus solely on the identification of tissue regions (e.g., stage I versus stage III ovarian tumors) where the number and/or type of detectable *N*-glycan species changes. The identification of these tissue regions will allow one of several additional experimental lines to be pursued. Firstly, the *N*-glycan profiles can be used to develop tissue-specific classifiers in a similar fashion to those developed for proteomic MALDI imaging profiles [14]. Secondly, the profiles could be used to guide glycoproteomics analyses where tryptic peptides are extracted, glycopeptides are enriched, and the mixture is analyzed by LC-MS/MS. Finally, further strategies could be developed which allow direct MALDI imaging of glycopeptides in a complex mixture of tryptic peptides, either through derivatization [36] or spatially sensitive enrichment. The straightforward methods presented here will allow the direct glycomic analysis of *N*-glycans in the vast archives of human cancer tissue which are available globally and ultimately increase our understanding of cancer biology through large retrospective studies.

## Electronic supplementary material

Below is the link to the electronic supplementary material.

ESM 1 (PDF 6857 kb)
