# Supplementary material for: MALDI imaging mass spectrometry of N-linked glycans on formalin-fixed paraffin-embedded murine kidney
Source: Anal Bioanal Chem. 2014 Dec 2;407(8):2127–39. doi: 10.1007/s00216-014-8293-7 (PMC4357650; doi:10.1007/s00216-014-8293-7)
Supplement: Supplementary file 1 — (PDF 6857 kb) [file 216_2014_8293_MOESM1_ESM.pdf]

Analytical and Bioanalytical Chemistry

Electronic Supplementary Material

MALDI imaging mass spectrometry of *N*-linked glycans on formalin-fixed murine kidney

Ove J.R. Gustafsson, Matthew T. Briggs, Mark R. Condina, Lyron J. Winderbaum, Matthias Pelzing, Shaun R. McColl, Arun V. Everest-Dass, Nicolle H. Packer, Peter Hoffmann

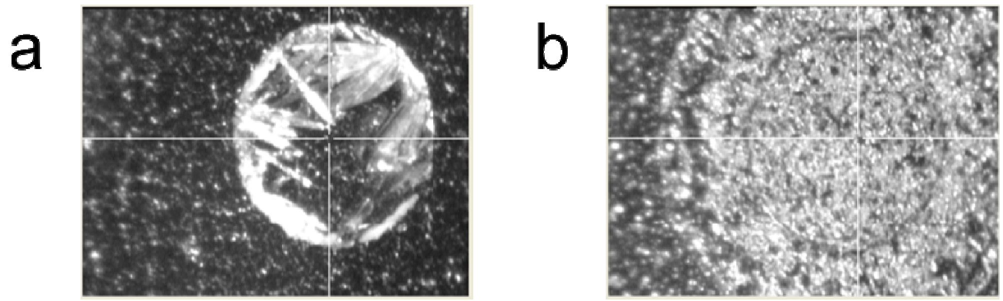

Fig. S1a and **b** dried droplet and EZYprep TM-sprayer 2,5-DHB matrix (10 mg/mL in 0.1% TFA and 1 mM NaCl) preparations respectively, as they appear on an MTP384-800µm AnchorChip solid sample support

Slide 1 section 1

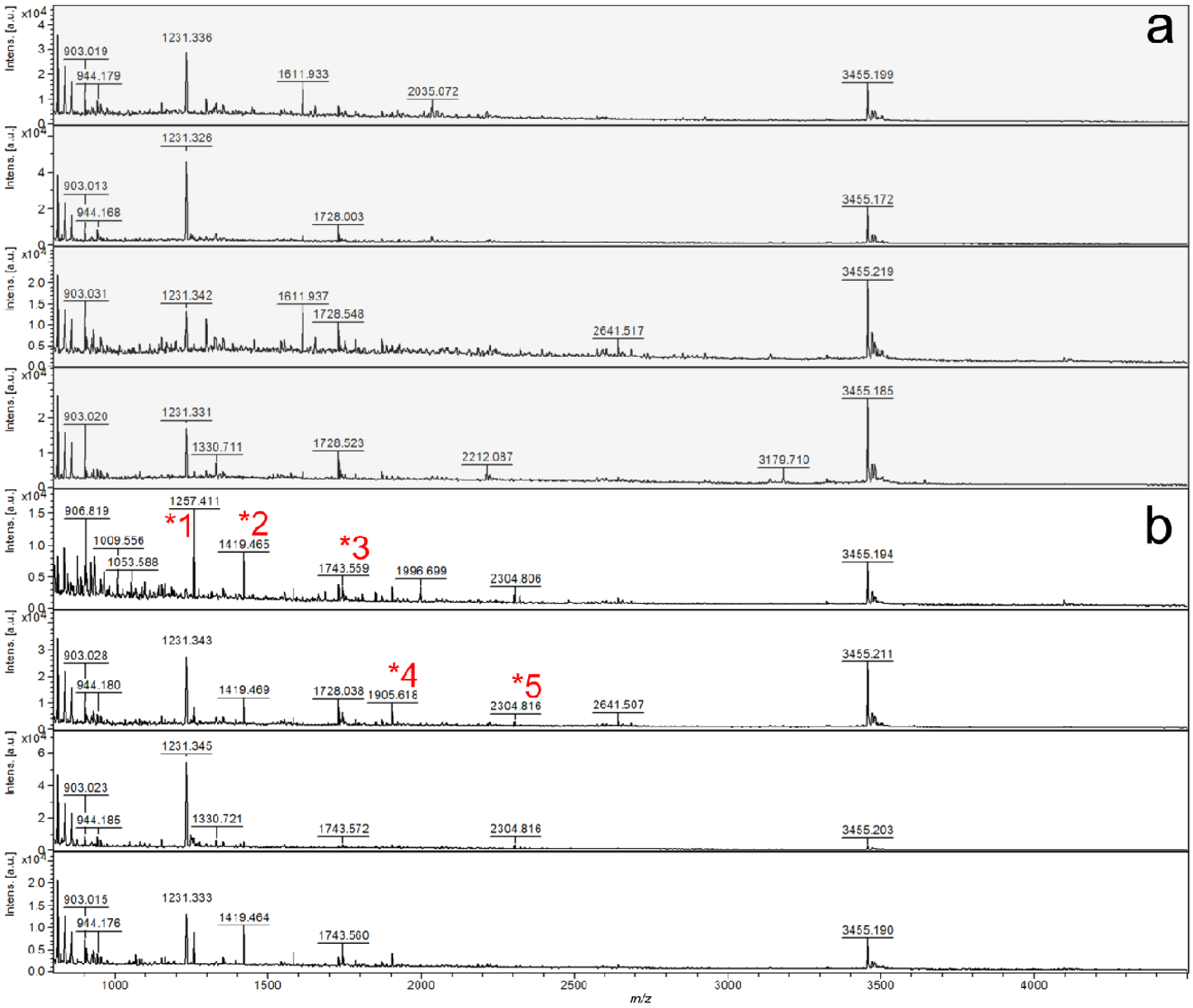

Fig. S2 *In situ* MALDI profiling MS spectra from tissue regions treated with buffer control **(a)** or PNGase F **(b)**

A 6 µm section (1 of N = 4) of formalin-fixed murine kidney was treated with citric acid antigen retrieval and subsequently subjected to ChIP-1000 deposition of 750 nL (total volume) of either buffer control (25 mM NH_4HCO_3) or PNGase F (in 25 mM NH_4HCO_3). Tissue was then incubated overnight at 37°C in a humid chamber. GLY3 calibrant (0.5 µL x 2 spots) was deposited onto a separate part of the same section. Tissue was overlaid with 10 mg/mL 2,5-DHB in 0.1% trifluoroacetic acid and 1 mM NaCl using a TM-sprayer instrument. MS spectra were acquired using an ultrafleXtreme MALDI-TOF/TOF instrument in reflectron positive ion mode

Slide 2 section 1

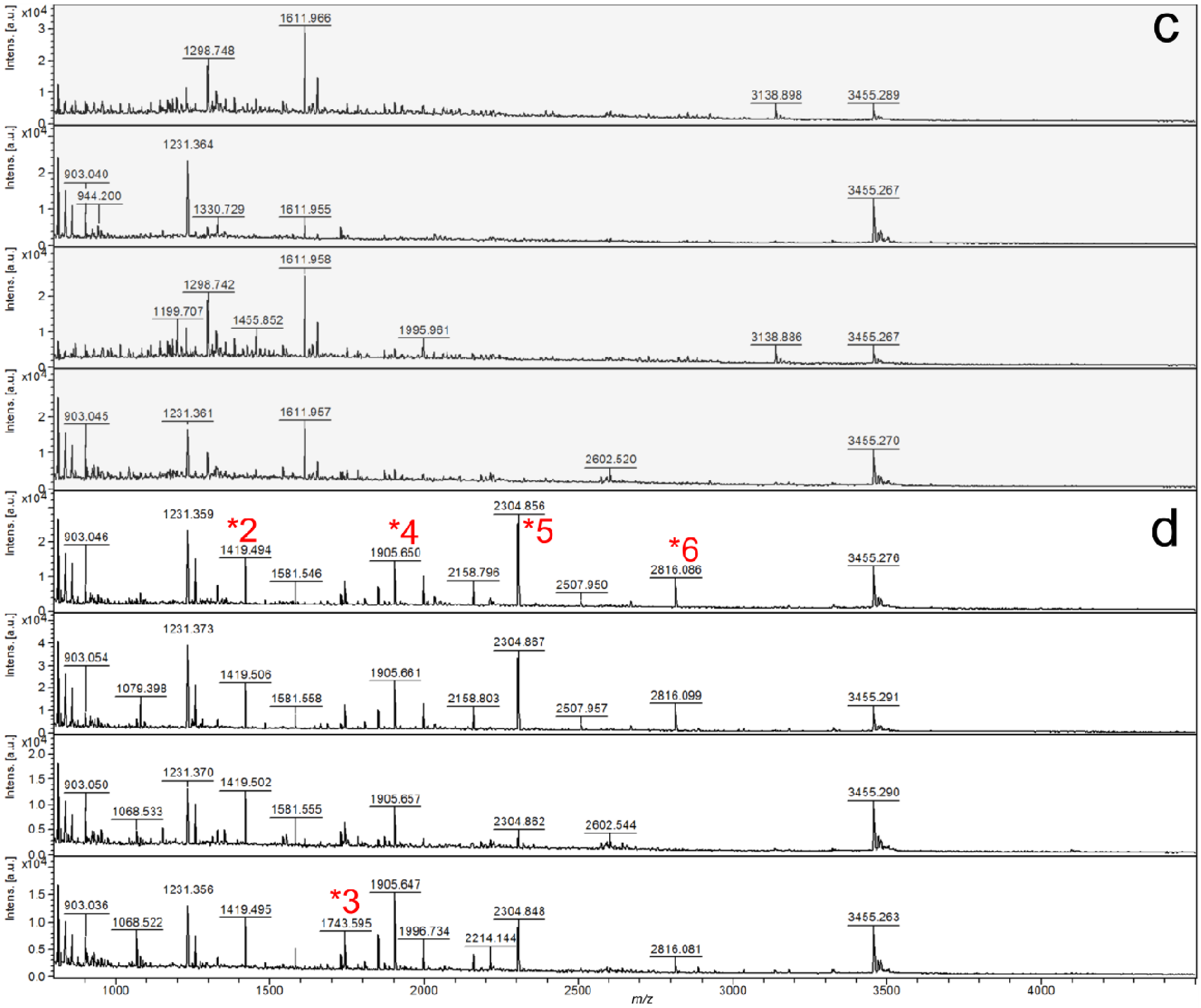

Fig. S2 *In situ* MALDI profiling MS spectra from tissue regions treated with buffer control **(c)** or PNGase F **(d)**

A 6 µm section (1 of N = 4) of formalin-fixed murine kidney was treated with citric acid antigen retrieval and subsequently subjected to ChIP-1000 deposition of 750 nL (total volume) of either buffer control (25 mM NH_4HCO_3) or PNGase F (in 25 mM NH_4HCO_3). Tissue was then incubated overnight at 37°C in a humid chamber. GLY3 calibrant (0.5 µL x 2 spots) was deposited onto a separate part of the same section. Tissue was overlaid with 10 mg/mL 2,5-DHB in 0.1% trifluoroacetic acid and 1 mM NaCl using a TM-sprayer instrument. MS spectra were acquired using an ultrafleXtreme MALDI-TOF/TOF instrument in reflectron positive ion mode

Slide 2 section 2

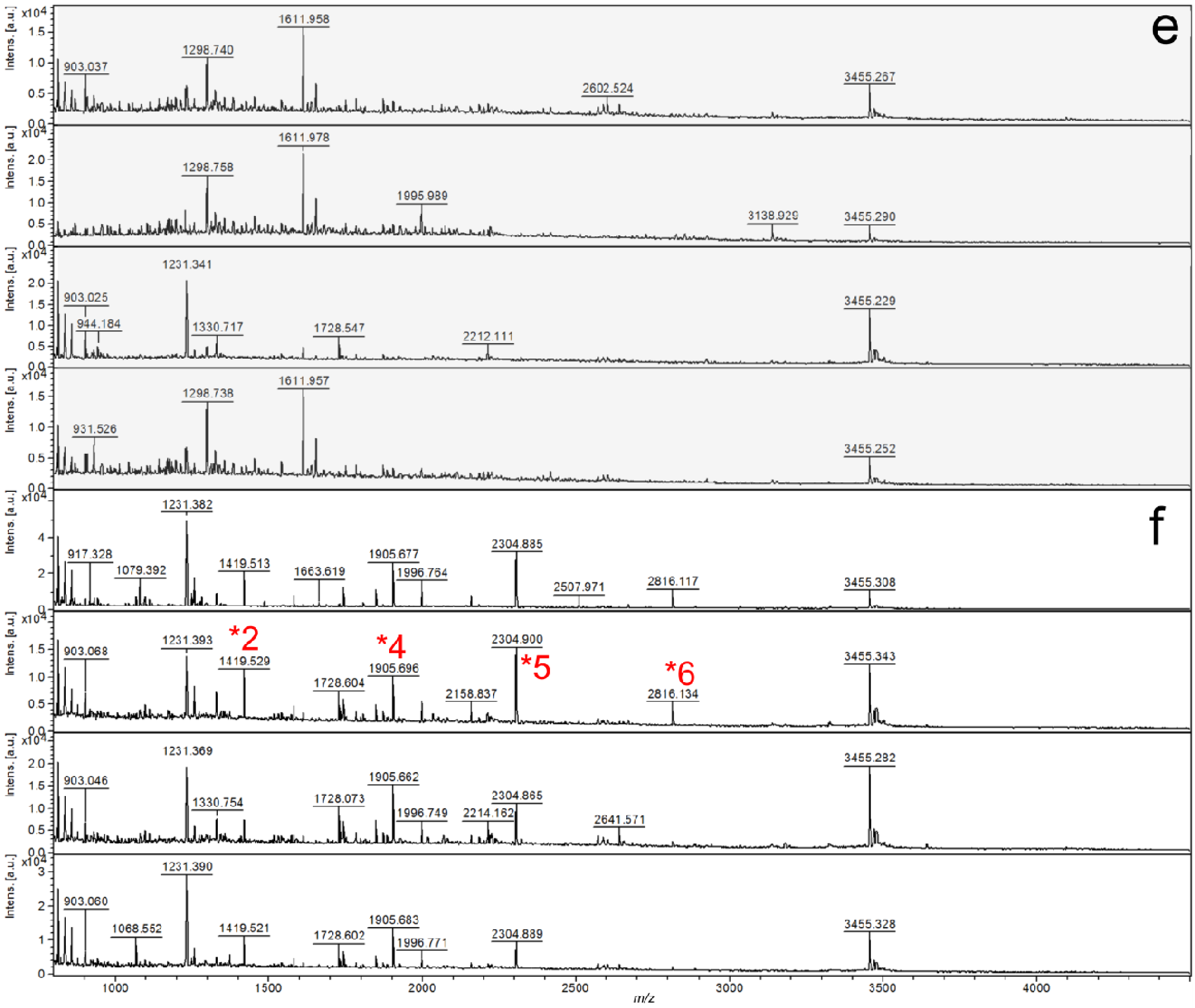

Fig. S2 *In situ* MALDI profiling MS spectra from tissue regions treated with buffer control **(e)** or PNGase F **(f)**

A 6 µm section (1 of N = 4) of formalin-fixed murine kidney was treated with citric acid antigen retrieval and subsequently subjected to ChIP-1000 deposition of 750 nL (total volume) of either buffer control (25 mM NH_4HCO_3) or PNGase F (in 25 mM NH_4HCO_3). Tissue was then incubated overnight at 37°C in a humid chamber. GLY3 calibrant (0.5 µL x 2 spots) was deposited onto a separate part of the same section. Tissue was overlaid with 10 mg/mL 2,5-DHB in 0.1% trifluoroacetic acid and 1 mM NaCl using a TM-sprayer instrument. MS spectra were acquired using an ultrafleXtreme MALDI-TOF/TOF instrument in reflectron positive ion mode

Table S1 N-glycans identified in a previous publication (Xu *et al.* 2012), as compared to the *in situ* large droplet profiling MS results obtained in the present study. Included are *m/z* calc. (theoretical calculated *m/z* – permethylated and sodiated) *m/z* det. (detected *m/z*) and N-glycan composition from Xu *et al.* (2012). Theoretical mass ([M]calc.) (from http://web.expasy.org/glycanmass/), theoretical sodiated mass ($[M+Na^+]^+$calc)., presence in profiling spectra, the measured *in situ* $[M+Na^+]^+$ and the error (ppm) between theoretical and measured $[M+Na^+]^+$ are also included

Xu et al. (2012)			*N-glycan composition*				*In situ MALDI profiling*			
x	*m/z calc. (permeth + Na⁺)*	*m/z det.*	*Hex*	*HexNAc*	*dHex*	*NeuAc*	*[M]calc.*	*$[M+Na^+]^+$calc.*	*Present in profiling*	*In situ $[M+Na^+]^+$*
1	1579.79	1579.92	5	2			1234.43	1257.42	Yes	1257.42
2	1783.89	1784.04	6	2			1396.49	1419.48	Yes	1419.48
3	1835.93	1836.09	3	4	1		1462.54	1485.53	Yes	1485.54
4	1906.97	1907.14	3	5			1519.57	1542.56	No	-
5	1987.99	1988.16	7	2			1558.54	1581.53	Yes	1581.54
6	2070.04	2070.2	5	4			1640.59	1663.58	Yes	1663.58
7	2081.06	2081.23	3	5	1		1665.62	1688.61	Yes	1688.61
8	2192.09	2192.27	8	2			1720.59	1743.58	Yes	1743.59
9	2244.13	2244.3	5	4	1		1786.65	1809.64	Yes	1809.64
10	2285.16	2285.36	4	5	1		1827.68	1850.67	Yes	1850.67
11	2396.19	2396.4	9	2			1882.64	1905.63	Yes	1905.64
12	2418.22	2418.43	5	4	2		1932.71	1955.70	Yes	1955.72
13	2459.25	2459.44	4	5	2		1973.73	1996.72	Yes	1996.73
14	2489.26	2489.46	5	5	1		1989.73	2012.72	Yes	2012.72
15	2530.28	2530.48	4	6	1		2030.76	2053.75	No	-
16	2592.31	2592.51	5	4	3		2078.77	2101.76	Yes	2101.79
17	2663.34	2663.55	5	5	2		2135.79	2158.78	Yes	2158.79
18	2674.36	2674.55	3	6	3		2160.82	2183.81	Yes	2183.84
19	2837.43	2837.62	5	5	3		2281.85	2304.83	Yes	2304.85

20	2867.44	2867.63	6	5	2		2297.84	2320.83	No	-
21	2908.47	2908.68	5	6	2		2338.87	2361.86	Yes	2361.88
22	3041.53	3041.69	6	5	3		2443.90	2466.89	No	-
23	3082.56	3082.76	5	6	3		2484.92	2507.91	Yes	2507.94
24	3112.53	3112.72	6	3		3	2472.85	2495.84	No	-
25	3112.57	3112.72	6	6	2		2500.92	2523.91	Yes	2523.92
26	3215.62	3215.76	6	5	4		2589.96	2612.95	No	-
27	2040.03	2040.2	4	4	1		1624.60	1647.59	Yes	1647.60
28	2111.07	2111.26	4	5			1681.62	1704.61	No	-
29	2162.08	2162.22	7	2	1		1704.60	1727.59	No	-
30	2326.18	2326.35	3	6	1		1868.70	1891.69	No	-
31	2377.19	2377.4	6	3	2		1891.68	1914.67	No	-
32	2448.23	2448.42	6	4	1		1948.70	1971.69	Yes	1971.73
33	2600.29	2600.48	10	2			2044.70	2067.69	Yes	2067.69
34	3183.61	3183.87	6	7	1		2557.94	2580.93	No	-

Table S2 Complete list of MALDI profiling AWM $[M+Na^{+}]^{+}$, the buffer control and PNGase F profiling counts as well as the calculated nLC-MS/MS $[M+Na^{+}]^{+}$, error in ppm, the doubly negatively charged calculated and measured masses ($[M-2H^{+}]^{2-}$ as well as the proposed composition and structure of identified N-glycans and the complete calculated $[M+Na^{+}]^{+}$ list from Xu *et al.* 2012

Buffer control count	PNGase F count	MALDI profiling AWM [M+Na+]+	LC-MS/MS [M+Na+]+ calculated	error (ppm)	$[M-2H]^{2-}$ calculated	$[M-2H]^{2-}$ measured	Proposed composition	Proposed structure	Xu *et al.* 2012
0	4	1009.56							
0	3	1025.59							
0	2	1028.10							
0	2	1048.17							
0	4	1053.59							
0	2	1067.60							
0	11	1068.52							
0	7	1079.38							
0	8	1089.61							
0	4	1095.37							
1	6	1111.53							
0	3	1141.64							
0	4	1164.75							
0	2	1185.66							
0	3	1229.68							
0	2	1246.34							
1	4	1247.35							
0	2	1251.31							
0	16	1257.42	1257.41	7.95	617.21	617.2	$(Hex)_2 + (Man)_3(GlcNAc)_2$	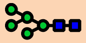	1257.42
0	2	1265.36							
0	5	1273.73							

0	2	1282.49							
0	2	1294.72							
1	4	1296.70							
0	3	1297.30							
0	2	1313.71							
0	3	1316.72							
			1339.47		658.24	658.2	$(HexNAc)_2$ + $(Man)_3(GlcNAc)_2$	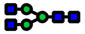	
1	3	1342.75							
1	4	1344.77							
0	2	1373.76							
0	3	1393.81							
0	2	1409.76							
0	16	1419.48	1419.47	7.04	698.24	698.3	$(Hex)_3$ + $(Man)_3(GlcNAc)_2$	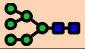	1419.48
0	6	1485.54	1485.53	6.73	731.27	731.3	$(HexNAc)_2$ $(Deoxyhexose)_1$ + $(Man)_3(GlcNAc)_2$	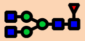	1485.53
			1542.55		759.78	759.7	$(HexNAc)_3$ + $(Man)_3(GlcNAc)_2$	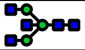	1542.56
1	16	1581.54	1581.53	6.32	779.27	779.3	$(Hex)_4$ + $(Man)_3(GlcNAc)_2$	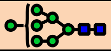	1581.53
0	3	1647.60	1647.57	18.21	812.29	812.3	$(Hex)_1$ $(HexNAc)_2$ $(Deoxyhexose)_1$ + $(Man)_3(GlcNAc)_2$	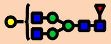	1647.59
0	9	1663.58	1663.57	6.01	820.29	820.3	$(Hex)_2$ $(HexNAc)_2$ + $(Man)_3(GlcNAc)_2$	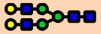	1663.58
0	10	1688.61	1688.61	0.00	832.81	832.8	$(HexNAc)_3$ $(Deoxyhexose)_1$ + $(Man)_3(GlcNAc)_2$	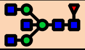	1688.61
			1704.59		840.8	840.8	$(Hex)_1$ $(HexNAc)_3$ + $(Man)_3(GlcNAc)_2$	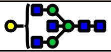	1704.61
									1727.59
0	2	1736.01							
1	4	1740.01							
1	4	1740.53							
0	16	1743.59	1743.57	11.47	860.29	860.3	$(Hex)_5$ + $(Man)_3(GlcNAc)_2$	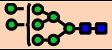	1743.58

			1767.59		872.3	872.3	$(Hex)_2$ $(HexNAc)_1$ $(NeuGc)_1$ + $(Man)_3(GlcNAc)_2$	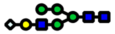	
0	3	1799.03							
0	12	1809.64	1809.63	5.25	893.32	893.4	$(Hex)_2$ $(HexNAc)_2$ $(Deoxyhexose)_1$ + $(Man)_3(GlcNAc)_2$	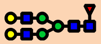	1809.64
			1809.63		893.32	893.4	$(Hex)_2$ $(HexNAc)_2$ $(Deoxyhexose)_1$ + $(Man)_3(GlcNAc)_2$	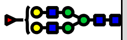	
0	15	1850.67	1850.65	10.81	913.83	913.9	$(Hex)_1$ $(HexNAc)_3$ $(Deoxyhexose)_1$ + $(Man)_3(GlcNAc)_2$	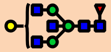	1850.67
									1891.69
1	16	1905.64	1905.63	5.25	941.32	941.3	$(Hex)_6$ + $(Man)_3(GlcNAc)_2$	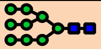	1905.63
									1914.67
0	2	1954.07	1954.67		965.84	965.8	$(Hex)_2$ $(HexNAc)_2$ $(NeuAc)_1$ + $(Man)_3(GlcNAc)_2$	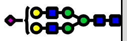	
0	2	1955.72	1955.69	15.34	966.35	966.3	$(Hex)_2$ $(HexNAc)_2$ $(Deoxyhexose)_2$ + $(Man)_3(GlcNAc)_2$	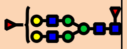	1955.70
			1970.67		973.84	973.8	$(Hex)_2$ $(HexNAc)_2$ $(NeuGc)_1$ + $(Man)_3(GlcNAc)_2$	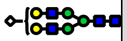	
0	2	1971.73							1971.69
			1995.69		986.35	986.4	$(Hex)_1$ $(HexNAc)_3$ $(NeuAc)_1$ + $(Man)_3(GlcNAc)_2$	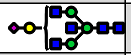	
0	16	1996.73							1996.72
0	6	2012.72	2012.71	4.97	994.86	994.8	$(Hex)_2$ $(HexNAc)_3$ $(Deoxyhexose)_1$ + $(Man)_3(GlcNAc)_2$	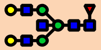	2012.72
0	2	2017.07							
0	2	2034.10							
									2053.75
1	2	2067.12							
0	6	2067.69	2067.67	9.67	1022.34	1022.4	$(Hex)_7$ + $(Man)_3(GlcNAc)_2$	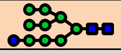	2067.69
0	2	2068.19							

			2100.73		1038.87	1038.9	$(Hex)_2$ $(HexNAc)_2$ $(Deoxyhexose)_1$ $(NeuAc)_1$ + $(Man)_3(GlcNAc)_2$	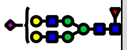	
0	2	2101.79	2101.75	19.03	1039.38	1039.4	$(Hex)_2$ $(HexNAc)_2$ $(Deoxyhexose)_3$ + $(Man)_3(GlcNAc)_2$	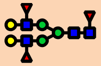	2101.76
			2116.73		1046.87	1046.9	$(Hex)_2$ $(HexNAc)_2$ $(Deoxyhexose)_1$ $(NeuGc)_1$ + $(Man)_3(GlcNAc)_2$	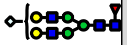	
0	3	2142.81							
0	10	2158.79	2158.77	9.26	1067.89	1067.8	$(Hex)_2$ $(HexNAc)_3$ $(Deoxyhexose)_2$ + $(Man)_3(GlcNAc)_2$	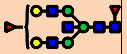	2158.78
0	2	2183.84							2183.81
1	8	2226.11							
			2303.81		1140.41	1140.4	$(Hex)_2$ $(HexNAc)_3$ $(Deoxyhexose)_1$ $(NeuAc)_1$ + $(Man)_3(GlcNAc)_2$	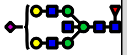	
1	15	2304.85	2304.83	8.68	1140.92	1140.9	$(Hex)_2$ $(HexNAc)_3$ $(Deoxyhexose)_3$ + $(Man)_3(GlcNAc)_2$	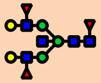	2304.83
									2320.83
0	6	2321.71							
1	2	2323.21							
0	4	2361.88							2361.86
			2391.83		1184.42	1184.4	$(Hex)_2$ $(HexNAc)_2$ $(Deoxyhexose)_1$ $(NeuAc)_2$ + $(Man)_3(GlcNAc)_2$	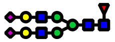	
			2407.81		1192.41	1192.4	$(Hex)_2$ $(HexNAc)_2$ $(Deoxyhexose)_1$ $(NeuAc)_1$ $(NeuGc)_1$ + $(Man)_3(GlcNAc)_2$	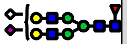	
			2423.81		1200.41	1200.4	$(Hex)_2$ $(HexNAc)_2$ $(Deoxyhexose)_1$ $(NeuGc)_2$ + $(Man)_3(GlcNAc)_2$	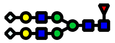	

									2466.89
									2495.84
0	7	2507.94							2507.91
0	3	2523.92							2523.91
									2580.93
1	4	2585.52							
									2612.95
1	5	2655.52							
0	7	2670.00							
1	5	2683.51							
0	9	2816.08	2816.01	24.86	1396.51	1396.6	$(Hex)_3$ $(HexNAc)_4$ $(Deoxyhexose)_4$ + $(Man)_3(GlcNAc)_2$	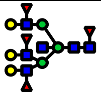	
0	6	2889.08							
0	5	3035.15							
1	2	3179.76							
0	6	3181.24							
0	5	3327.35							
0	7	3502.26							
1	3	4096.66							

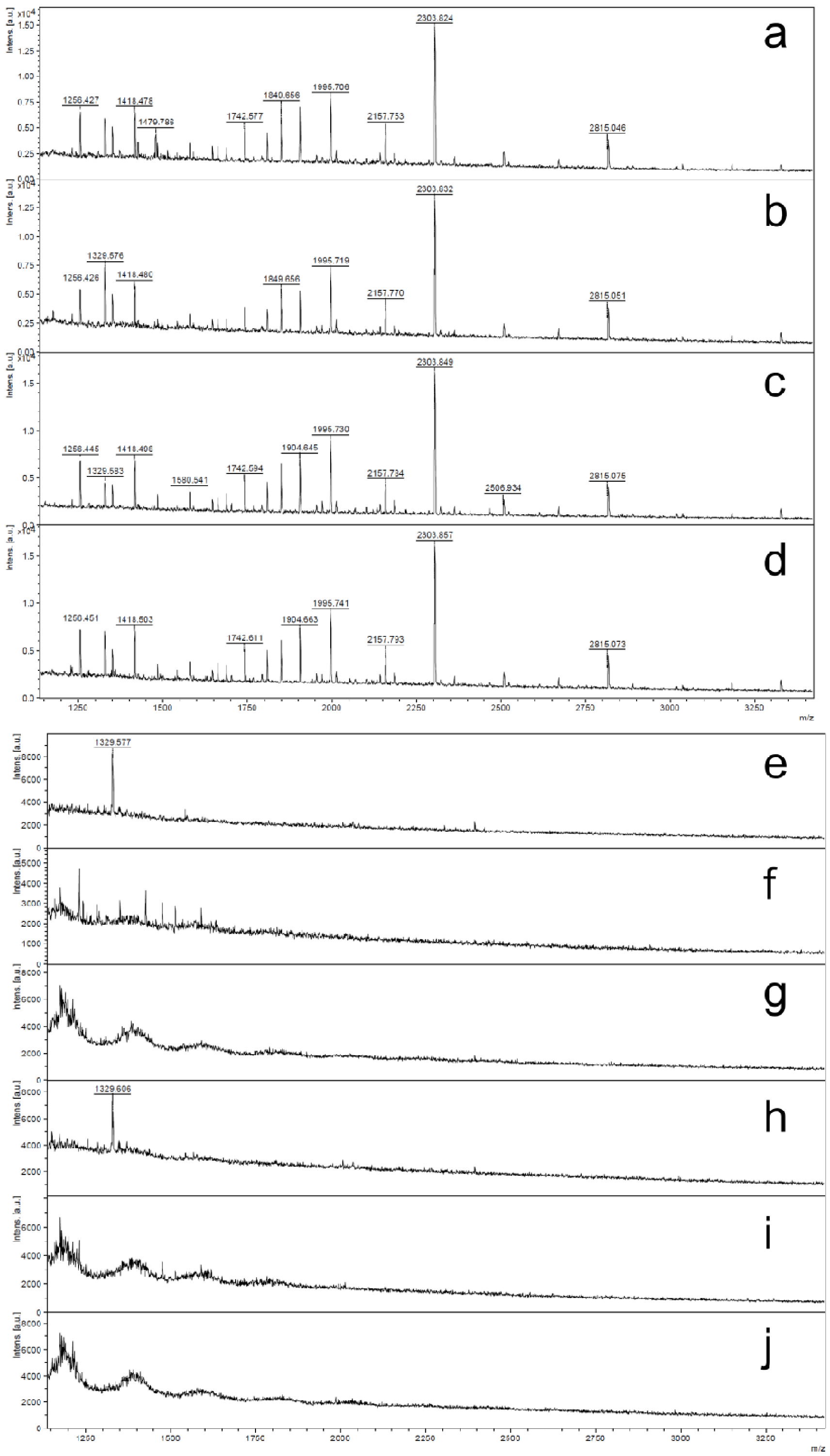

Fig. S3 In-solution formalin-fixed tissue PNGaseF treatment and on-target MALDI-TOF/TOF MS results, with controls

Six µm sections of formalin-fixed paraffin-embedded murine kidney were treated using four conditions, in solution and in duplicate. Following citric acid antigen retrieval, 10 mM NH_4HCO_3 washes were used to remove citric acid. The conditions were as follows: tissue and PNGaseF (**a, b, c, d**), PNGaseF only (**f, i**), tissue only (**e, h**) and no tissue or PNGaseF (**g, j**). Total reaction volumes were 40 µL. PNGaseF volumes were constant at 2 µL (1000 NEB units) and buffer used was 25 mM NH_4HCO_3. All reactions were kept at 37°C overnight. Sample and GLY3 calibrant spotted onto an AnchorChip target and allowed to dry were overlaid with 10 mg/mL 2,5-DHB in 0.1% trifluoroacetic acid and 1 mM NaCl using a TM-sprayer. MS spectra were acquired on an ultrafleXtreme MALDI-TOF/TOF instrument in reflectron positive ion mode

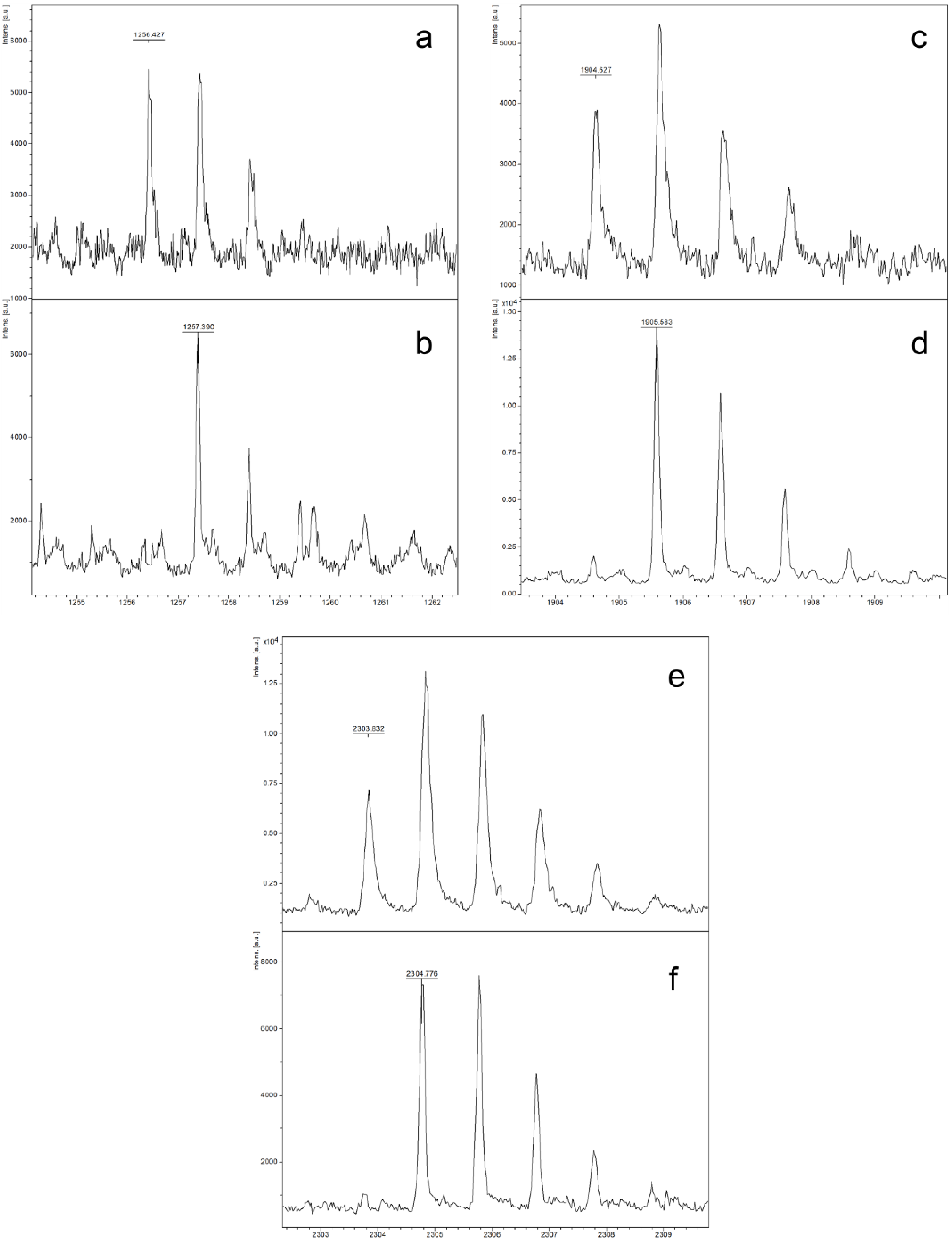

Fig. S4 Isotopic profiles of on target and *in situ* MS

Panels **a,c** and **e** show the MALDI-TOF/TOF MS spectra of N-glycans from in-solution PNGase F treated tissue sections, spotted onto an AnchorChip solid sample support and overlaid with 2,5-DHB matrix. Panels **b, d** and **f** show the MALDI-TOF/TOF MS spectra from direct analysis of tissue sections treated with PNGase F and overlaid with 2,5-DHB matrix

Fig. S5 Fragmentation spectra for three N-glycans

a) *m/z* 941.0 $(Hex)_6$+$(Man)_3(GlcNAc)_2$,
b) *m/z* 1038.8 $(Hex)_2(HexNAc)_2(Deoxyhexose)_1(NeuAc)_1$ + $(Man)_3(GlcNAc)_2$
c) *m/z* 1039.3 $(Hex)_2$ $(HexNAc)_2$ $(Deoxyhexose)_3$ + $(Man)_3(GlcNAc)_2$

N-glycan mixture was reduced, cleaned on carbon columns and fractionated by nLC on a graphite column for analysis by Ion Trap MS and MS/MS.

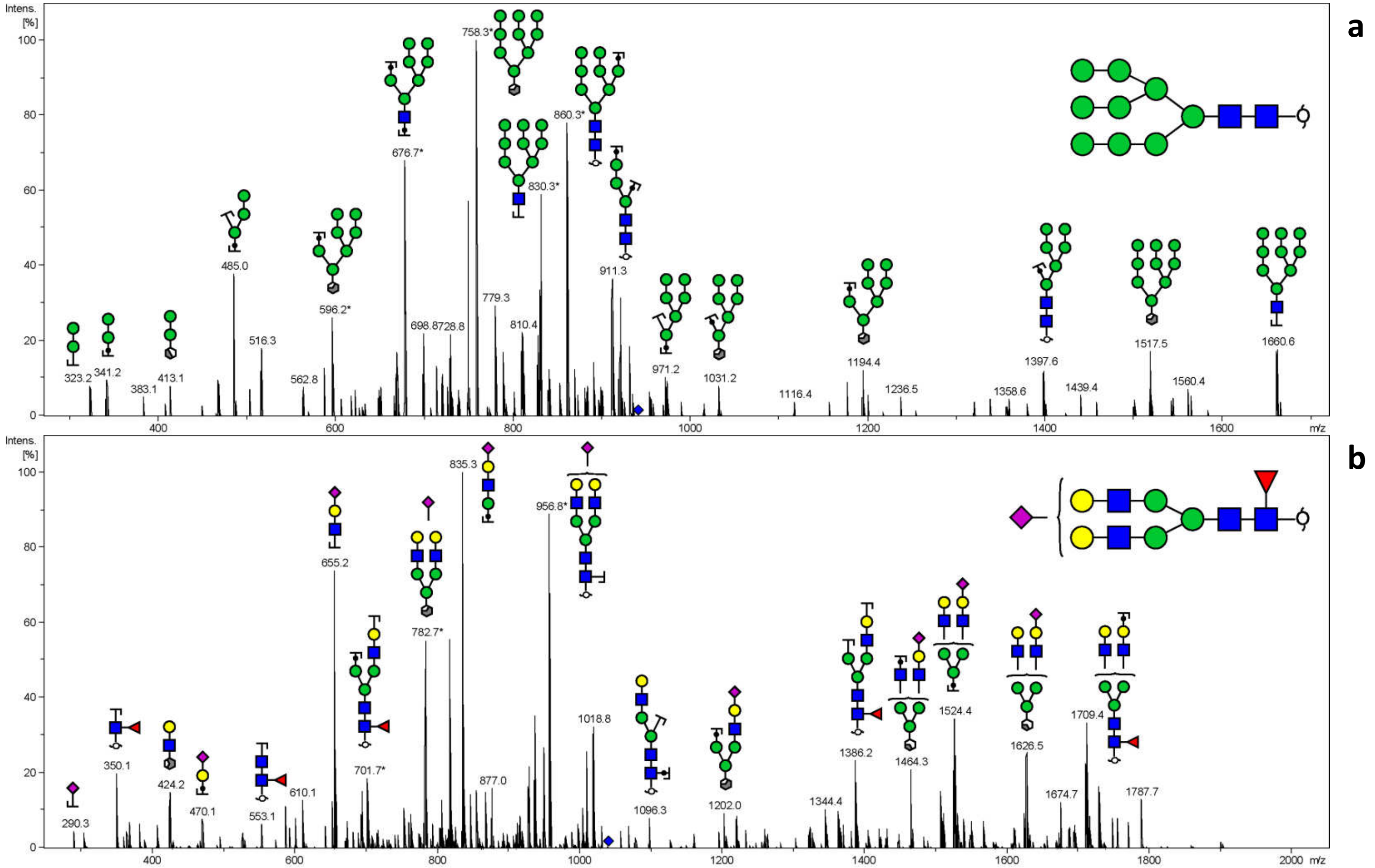

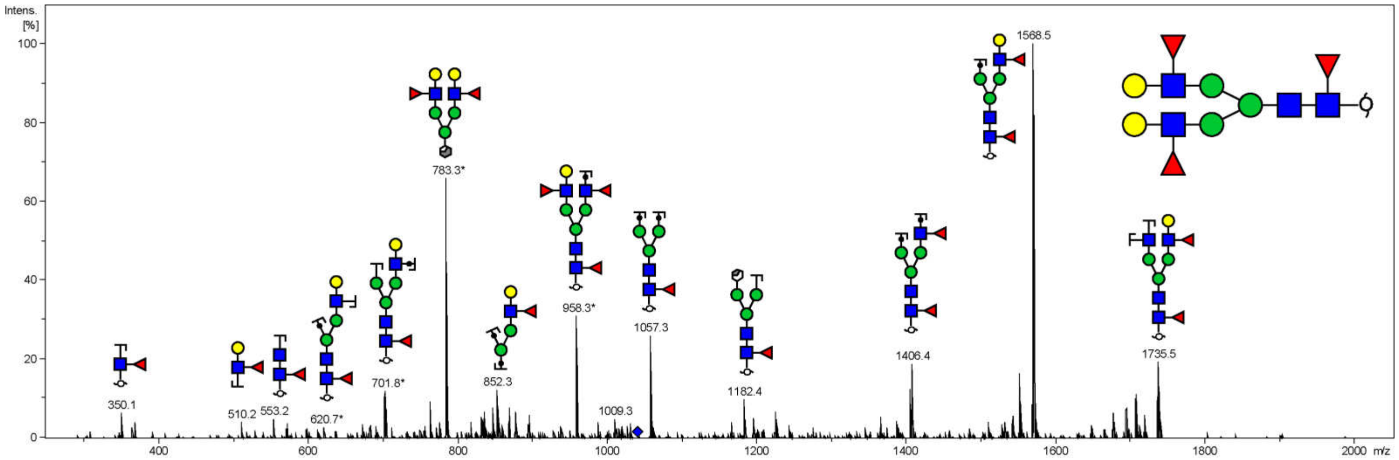

Intens.
[%]
100
80
60
40
20
0
400
600
800
1000
1200
1400
1600
1800
2000 m/z
350.1
510.2
553.2
620.7*
701.8*
783.3*
852.3
958.3*
1009.3
1057.3
1182.4
1406.4
1568.5
1735.5

c

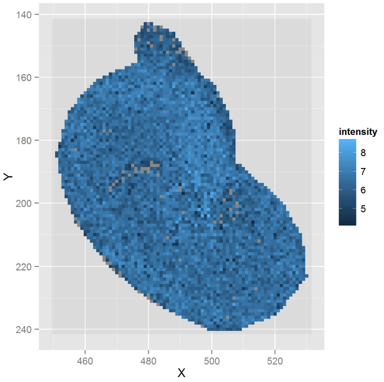

a. 1257.473

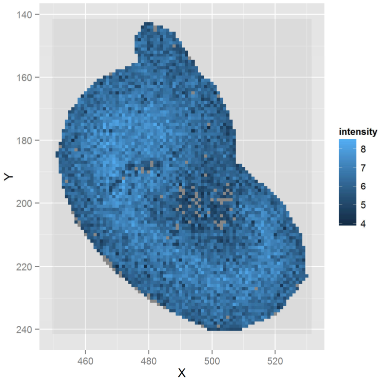

b. 1905.697

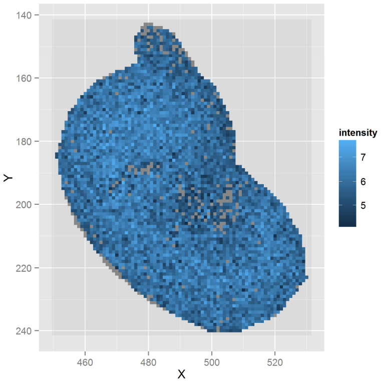

c. 1743.643

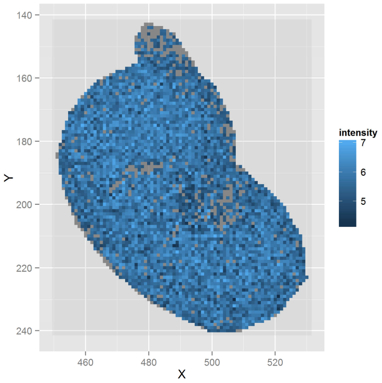

d. 1581.587

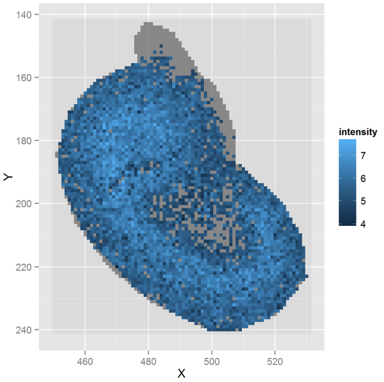

e. 1850.730

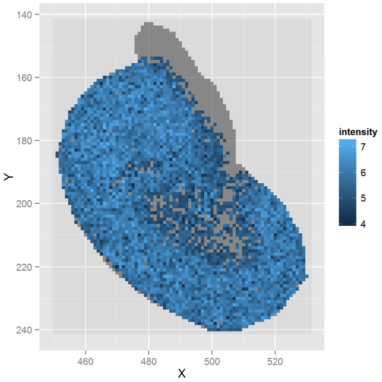

f. 1996.788

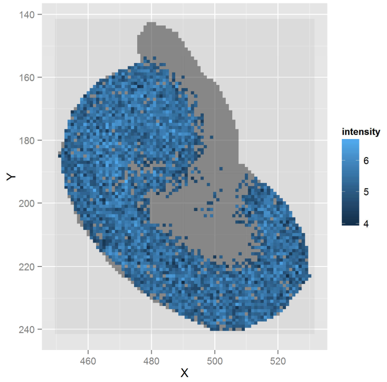

g. 2158.844

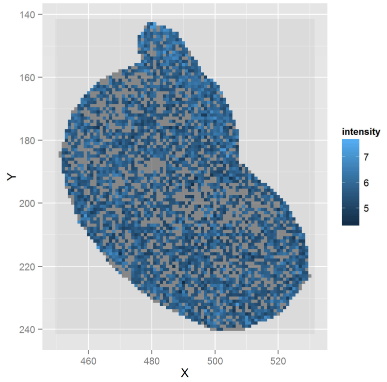

h. 822.944

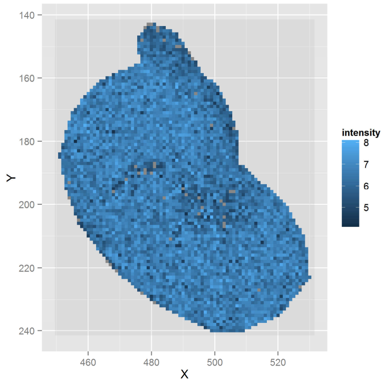

i. 1419.532

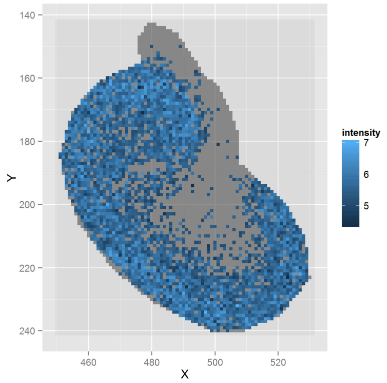

j. 917.345

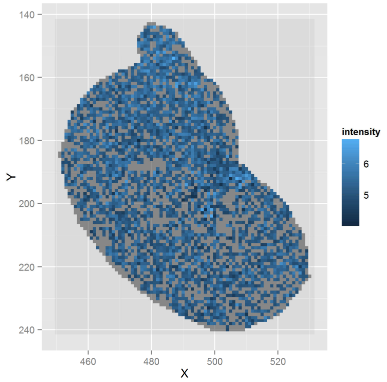

k. 1809.690

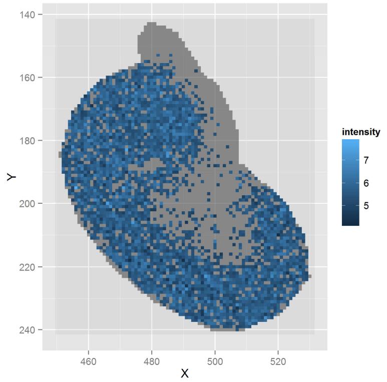

l. 1079.408

Fig. S6 Log ion intensity maps for *m/z* values above the DIPPS cut-off threshold for N-glycan MALDI imaging

Panels **a-k** show the log ion intensity maps for 12 *m/z* values assigned as important (based on occurrence through DIPPS analysis) to the PNGase F treated tissue sections, as compared to buffer control
